# Detection of Clinically Significant Drug-Drug Interactions in Fatal Torsades de Pointes: Disproportionality Analysis of the Food and Drug Administration Adverse Event Reporting System

**DOI:** 10.2196/65872

**Published:** 2025-03-25

**Authors:** Huanhuan Ji, Meiling Gong, Li Gong, Ni Zhang, Ruiou Zhou, Dongmei Deng, Ya Yang, Lin Song, Yuntao Jia

**Affiliations:** 1 Department of Pharmacy National Clinical Research Center for Child Health and Disorders, Ministry of Education Key Laboratory of Child Development and Disorders, China International Science and Technology Cooperation base of Child development and Critical Disorders Children’s Hospital of Chongqing Medical University Chongqing China; 2 School of Pharmacy Chongqing Medical University Chongqing China; 3 Department of Phase I Clinical Trial Ward Chongqing Key Laboratory of Translational Research for Cancer Metastasis and Individualized Treatment Chongqing University Cancer Hospital Chongqing China; 4 Department of Pharmacy The Daping Hospital of Army Medical University Chongqing China

**Keywords:** torsades de pointes, FAERS database, drug-drug interactions, QTc-prolonging drugs, adverse drug events

## Abstract

**Background:**

Torsades de pointes (TdP) is a rare yet potentially fatal cardiac arrhythmia that is often drug-induced. Drug-drug interactions (DDIs) are a major risk factor for TdP development, but the specific drug combinations that increase this risk have not been extensively studied.

**Objective:**

This study aims to identify clinically significant, high-priority DDIs to provide a foundation to minimize the risk of TdP and effectively manage DDI risks in the future.

**Methods:**

We used the following 4 frequency statistical models to detect DDI signals using the Food and Drug Administration Adverse Event Reporting System (FAERS) database: Ω shrinkage measure, combination risk ratio, chi-square statistic, and additive model. The adverse event of interest was TdP, and the drugs targeted were all registered and classified as “suspect,” “interacting,” or “concomitant drugs” in FAERS. The DDI signals were identified and evaluated using the Lexicomp and Drugs.com databases, supplemented with real-world data from the literature.

**Results:**

As of September 2023, this study included 4313 TdP cases, with 721 drugs and 4230 drug combinations that were reported for at least 3 cases. The Ω shrinkage measure model demonstrated the most conservative signal detection, whereas the chi-square statistic model exhibited the closest similarity in signal detection tendency to the Ω shrinkage measure model. The κ value was 0.972 (95% CI 0.942-1.002), and the *P_positive_* and *P_negative_* values were 0.987 and 0.985, respectively. We detected 2158 combinations using the 4 frequency statistical models, of which 241 combinations were indexed by Drugs.com or Lexicomp and 105 were indexed by both. The most commonly interacting drugs were amiodarone, citalopram, quetiapine, ondansetron, ciprofloxacin, methadone, escitalopram, sotalol, and voriconazole. The most common combinations were citalopram and quetiapine, amiodarone and ciprofloxacin, amiodarone and escitalopram, amiodarone and fluoxetine, ciprofloxacin and sotalol, and amiodarone and citalopram. Although 38 DDIs were indexed by Drugs.com and Lexicomp, they were not detected by any of the 4 models.

**Conclusions:**

Clinical evidence on DDIs is limited, and not all combinations of heart rate–corrected QT interval (QTc)–prolonging drugs result in TdP, even when involving high-risk drugs or those with known risk of TdP. This study provides a comprehensive real-world overview of drug-induced TdP, delimiting both clinically significant DDIs and negative DDIs, providing valuable insights into the safety profiles of various drugs, and informing the optimization of clinical practice.

## Introduction

Torsades de pointes (TdP) is a rare but life-threatening cardiac arrhythmia characterized by a prolonged heart rate–corrected QT interval (QTc) on an electrocardiogram. TdP is often induced by QTc-prolonging drugs, which can further increase the risk when used in combination. According to the Arizona Center for Education and Research on Therapeutics, 268 drugs are known to be QTc-prolonging drugs, including antiarrhythmics, antibiotics, antipsychotics, antidepressants, and antineoplastic drugs. These drugs are classified into 3 categories: 68 drugs with a known risk of TdP, 148 drugs with a possible risk of TdP, and 52 drugs with a conditional risk of TdP [1]. Chien et al [2] conducted a large-scale, case-crossover study demonstrating that QT-prolonging drug use and related drug-drug interactions (DDIs), particularly “known risk” or “conditional risk” drugs, are significantly associated with QTc prolongation. This risk is further exacerbated by the concurrent administration of these drugs with other QT-prolonging agents or potent cytochrome P450 inhibitors. The resulting exponential increase in potential DDIs is considerable and warrants careful consideration when these medications are used concurrently. In clinical settings, DDI alerts are implemented to warn of potential QTc DDI risks. However, their unclear clinical relevance often leads to indiscriminate alerts, resulting in over 90% being ignored, limiting the effectiveness of QTc DDI alerts [3,4]. Wasserman et al [4] found an average of 3 unique QTc DDI alerts presented per patient admission (range 1-44), with an average of 6 overridden QTc DDI alerts (range 1-432), ultimately resulting in 29% of admissions experiencing a drug-induced QTc prolongation with a ≥60-point increase in QTc value from baseline or a post-QTc value of ≥500 ms, representing a significant increase in TdP risk. Although DDIs are recognized as a significant risk factor for TdP development, the specific drug combinations contributing to this risk remain poorly characterized.

In clinical practice, the assessment of DDI risks often relies on multiple information sources, such as Micromedex, UpToDate, Drugs.com, and Medscape.com. However, significant variability exists among these resources, as demonstrated by Shariff et al [5], who reported poor intersource reliability scores (κ<0.20, *P*<.05) [5]. This inconsistency complicates decision-making for health care professionals and underscores the importance of using real-world data to detect clinically significant DDIs. The challenge is particularly acute with older adults, patients with cancer, and critically ill patients with multiple comorbidities, who often receive complex drug regimens that extend beyond the 2-drug combinations typically considered in DDI reports [6-8].

Literature data serve as valuable real-world evidence, with numerous cases of drug-induced TdP documented. However, there is a notable paucity of evidence concerning DDIs in these reports. Krumpholz et al [9] conducted a comprehensive review of literature-reported cases of drug-induced TdP from 1980 to 2021, encompassing 424 papers of 634 case reports, which primarily documented suspected and concomitant drugs without adequately considering interacting drugs. This highlights the need for studies focusing on identifying clinically significant DDIs.

The Food and Drug Administration Adverse Event Reporting System (FAERS) database is a spontaneous reporting system that provides information about adverse events (AEs) in clinical settings and has emerged as a valuable resource for pharmacovigilance research. Although the database has limitations, including potential inaccuracies, underreporting, and missing data, it provides unique insights into postmarketing drug safety. In the FAERS database, 2 or more suspected, interacting, or concomitant drugs for each event can be given, which is particularly useful for identifying DDIs that result in severe or fatal AEs. However, significant uncertainty exists regarding the association between drugs and AEs in the given report, as these reports encompass all potential drugs and AEs for a patient. Recent methodological advances have enhanced the utility of FAERS for DDI detection, particularly through the application of multiple statistical models that can improve signal detection reliability [10,11]. This study aimed to integrate multiple data sources, including the FAERS database, DDI information resources, and relevant literature, to detect clinically significant DDIs associated with TdP risk. By using advanced analytical methods and cross-validating findings across multiple sources, we sought to provide more reliable and actionable insights for clinical decision-making.

## Methods

### Data Sources

A retrospective, disproportionality, pharmacovigilance study was conducted from the first quarter of 2004 to the second quarter of 2023 using the FAERS database to detect potential DDIs that increase the incidence of TdP in a large-scale population. In the FAERS database, each report is coded using the preferred terms (PTs) provided by the Medical Dictionary for Regulatory Activities (MedDRA), and each drug is assigned a code according to its association with AEs: suspect drugs, concomitant drugs, or interacting drugs. OpenVigil 2.1-MedDRA-v24 [12], an open tool for data mining and analysis of pharmacovigilance data using cleansed FDA AE reporting data, was used to detect DDIs.

### Definition of AE

In this study, the targeted AE was TdP, which was extracted from FAERS using the PT of TdP (PT code: 10044066). In these cases, all drugs classified as suspected drugs, interacting drugs, and concomitant drugs were included in the process to investigate DDIs. For signal detection, we focused only on combinations of 2 drugs with 3 or more cases.

### Suspect Drugs of TdP Cases

Single-drug TdP signals were assessed for disproportionality using the established pharmacovigilance index, the reporting odds ratio (ROR). ROR values were calculated as (a × d)/(b × c) and expressed as point estimates with a 95% CI. In our study, an event was considered significant when the ROR value was greater than 2 and the lower limit of the 95% CI of the ROR was greater than 1, and at least 3 cases were required to define a positive signal for the ROR.

### DDI Signal Detection Statistical Models and Standards

Several statistical algorithms have been developed for exploring DDI signals, yet no de facto standard exists for DDI signal detection in spontaneous reporting systems [10,11]. Each method has inherent advantages and limitations. Among them, the Ω shrinkage measure model, proposed by Norén et al [13] and used by the World Health Organization Uppsala Monitoring Center, calculates an observed-to-expected ratio for disproportionality measurement of potential DDIs. This method demonstrates a conservative signal detection trend among frequentist-based approaches, effectively minimizing false positives by handling sparse data, adjusting for deviations in expected frequencies, and shrinking the extreme ratios toward the overall mean. It is particularly well-suited for monitoring rare AEs (eg, TdP) in noisy databases like FAERS but requires clinical validation to confirm associations between drug combinations and adverse reactions. The additive model, as described by Thakrar et al [14], estimates co-medication risks by evaluating target AE incidences across different exposure scenarios. Although highly sensitive for detecting potential drug synergies, it is prone to spurious associations due to noise and biases in spontaneous reporting systems. Similarly, the chi-square statistic model with Yates correction, proposed by Gosho et al [15], is widely used but vulnerable to false negatives or false positives when applied to sparse data, limiting its utility for rare events like TdP. The combination risk ratio model, proposed by Noguchi et al [16], offers a theoretical framework for assessing the joint risk of co-medicated drugs by assuming that the occurrence of AEs represents a combined risk of both drugs. This approach is valuable for exploring potential interactions but relies heavily on sufficient co-medication frequency, limiting its effectiveness with rare drug combinations.

To address these limitations, a multimethod cross-validation approach was used, integrating the Ω shrinkage measure model, additive model, chi-square statistic model, and the combination risk ratio model (calculation methods are detailed in Multimedia Appendix 1). This ensures that signals detected by multiple methods are less likely to be statistical artifacts. Ω shrinkage was prioritized as the baseline model due to its robustness at handling sparse data and its ability to minimize false positives. Additive models were further screened using Ω shrinkage to reduce spurious associations. For signal confirmation, all 4 statistical models were applied, with a positive signal defined as one consistently detected by all 4 methods. We constructed 2 contingency tables—a 4-by-2 table and a 2-by-2 table—to facilitate the analysis (detailed in Multimedia Appendix 1). Additionally, the specific criteria for signal detection applied to each model are detailed in Table 1.

**Table 1:** The signal detection criteria for each statistical model.

Statistical models	Criteria for positive signals
Ω shrinkage measure model	Ω_025_ > 0, *n*_111_ ≥ 3
Chi-square statistic model	χ > 2, *n*_111_ ≥ 3
Combination risk ratio model	*PRR*^a^_*drug D*__1 ∩__*drug D*__2_ > 2, χ^2^_*drug D*__1 ∩__*drug D*__2_ > 4, *CRR*^b^ > 2, *n*_111_ ≥ 3
Additive model	*P*_*11*_ – *P*_*10*_ –*P*_*01*_ + *P*_*00*_ >0, *n*_111_ ≥ 3

^a^PRR: proportional reporting ratio.

^b^CRR: combination risk ratio.

To ensure robustness and minimize false positives, positive DDI signals underwent external validation by cross-referencing with established DDI databases and a comprehensive literature review of relevant case reports to verify their clinical significance.

### Evaluation of Commonality of Signals Detected

The commonality of the signals detected by each statistical model was evaluated using the Cohen kappa coefficient (κ), the proportionate agreement for positive rating (*P_positive_*), and the proportionate agreement for negative rating (*P_negative_*), as reported in a previous study (calculation methods are detailed in Multimedia Appendix 1). The κ provides a measure that adjusts the observed agreement for the chance agreement, which ranges from –1 to 1, and indicates the level of agreement between observed and expected values [16]. A κ value of 0 to 0.20 indicates slight agreement, 0.21 to 0.4 indicates fair agreement, 0.41 to 0.60 indicates moderate agreement, 0.61 to 0.80 indicates substantial agreement, 0.81 indicates upward excellent agreement, and the absolute agreement is 1 [17]. Conversely, κ<0 means that the observed agreement rate is less than the chance agreement rate, which rarely occurs in practical research.

### Signal Verification

As positive controls, we downloaded a list of 268 drugs with known (n=68), possible (n=148), and conditional (n=52) risks of TdP from CredibleMed, an online compendium of QTc drugs [1], and 217 drugs with the highest (n=32), moderate (n=70), and low (n=115) risks of interacting from UpToDate [18].

To ensure the accuracy and reliability of DDI results, the Lexicomp point-of-care database, known for its comprehensive scope, was used to validate DDI signals. Additionally, the open-access online drug interaction checker Drugs.com was used to cross-validate the DDI signals. The severity classifications of DDIs in the 2 drug databases are detailed in Multimedia Appendix 1 [18,19]. Subsequently, a search was conducted in the PubMed database for literature pertaining to drug-induced TdP, and an open-access database of literature-derived drug-related TdP cases, containing 624 TdP cases from 424 papers, was used to further evaluate the clinical relevance of the DDIs [9].

### Sensitivity Analyses

Given that nonprofessional reporters may not have the expertise to accurately distinguish between AEs and the symptoms of the disease itself, this could potentially affect the interpretability of aggregated results, particularly in large-scale data sets like FAERS. To improve the clarity and reliability of the findings, we excluded data reported by nonprofessionals from the validated positive signal data, and conducted sensitivity analysis to assess robustness.

### Ethical Considerations

This study uses the publicly accessible and anonymized FAERS database, which contains no personally identifiable information and does not involve human subject experimentation. As a result, it is exempt from review and approval by an institutional review board.

## Results

### Basic Information on TdP Cases

As of September 2023, a total of 4313 TdP cases from 11,439,756 AE cases were included in this study. Of the 4313 TdP cases, 2403 (55.7%) were female, and 1398 (32.4%) were male. The average age was 55.5 (range 0-97) years, and 1653 (38.3%) were older than 60 years. TdP is serious and fatal, with many of the reported cases leading to hospitalization (initial or prolonged; n=1310, 30.4%), life-threatening events (n=1705, 39.5%), or death (n=363, 8.4%). Notably, reports submitted by nonprofessionals, such as consumers and lawyers, accounted for only 290 (6.7%) of the total. This proportion is significantly lower than the 20% to 30% typically reported, likely due to the rarity and severity of TdP, which often requires hospitalization, thereby limiting the likelihood of reports from consumers. Geographically, 1784 (41.4%) of these 4313 cases originated from the United States (Table 2).

**Table 2:** Characteristics of patients with torsades de pointes in the Food and Drug Administration Adverse Event Reporting System database (n=4313).

Characteristic			Patients, n (%)
**Gender**			
	Male	1398 (32.4)	
	Female	2403 (55.7)	
	Unknown	512 (11.9)	
**Age (years)**			
	0-19	180 (4.2)	
	20-39	625 (14.5)	
	40-59	1011 (23.4)	
	60-79	1196 (27.7)	
	≥80	457 (10.6)	
	Unknown	844 (19.6)	
**Severity**			
	Died	363 (8.4)	
	Life threatening	1705 (39.5)	
	Disabled	6 (0.1)	
	Hospitalized: initial or prolonged	1310 (30.4)	
	Required intervention	19 (0.4)	
	Other outcomes	882 (20.4)	
	Unknown	28 (0.6)	
**Reporter**			
	Other health professionals^a^	1638 (38)	
	Medical doctor	1585 (36.7)	
	Pharmacist	467 (10.8)	
	Not obtained	333 (7.7)	
	Consumer	218 (5.1)	
	Lawyer	72 (1.7)	
**Reporter country**			
	United States	1784 (41.4)	
	Japan	201 (4.7)	
	China	80 (1.9)	
	Other countries	1923 (44.6)	
	Unknown	325 (7.5)	

^a^Other health professionals included 1 registered nurse and 396 health professionals.

**Figure 1:**
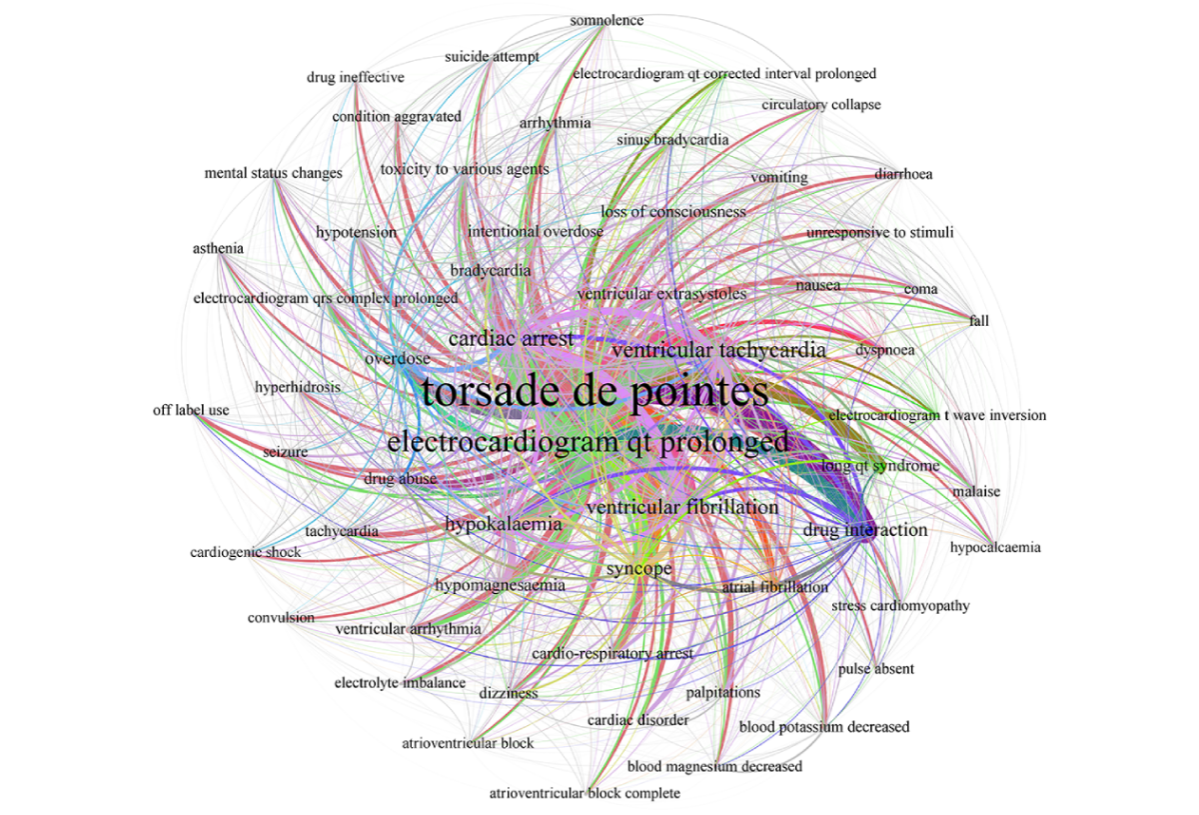
Cocurrent network diagram of adverse events related to torsades de pointes.

Figure 1 depicts a cocurrent network of AEs associated with TdP, providing a comprehensive overview of the patterns of TdP occurrence. The size of a node is proportional to the number of neighboring AEs, while the width of an edge is proportional to the number of unique AEs. Among the 4313 TdP cases analyzed, the most common presentations were electrocardiogram QT prolonged (n=1736), long QT syndrome (n=298), electrocardiogram QT-corrected interval prolonged (n=61), cardiac arrest (n=892), ventricular tachycardia (n=738), ventricular fibrillation (n=622), syncope (n=492), ventricular extrasystoles (n=224), loss of consciousness (n=214), cardiorespiratory arrest (n=205), atrial fibrillation (n=144), arrhythmia (n=140), ventricular arrhythmia (n=135), tachycardia (n=115), palpitations (n=114), and dizziness (n=109). The risk factors of hypokalemia, hypomagnesemia, bradycardia, electrolyte imbalance, decreased blood magnesium, and hypocalcemia were observed in 524, 235, 225, 65, 63, and 50 cases, respectively. The drug-related risk factors of drug interactions, overdose, drug abuse, intentional overdose, and toxicity to various agents were present for 513, 271, 168, 164, and 162 cases, respectively.

**Figure 2:**
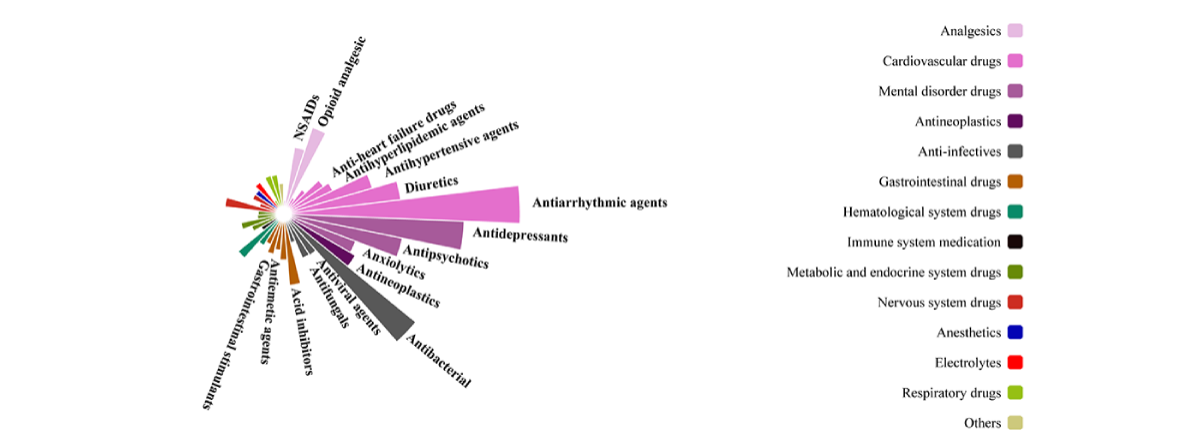
Distribution of torsades de pointes–related drug classes.

### Suspect Drugs of TdP Cases

**Figure 3:**
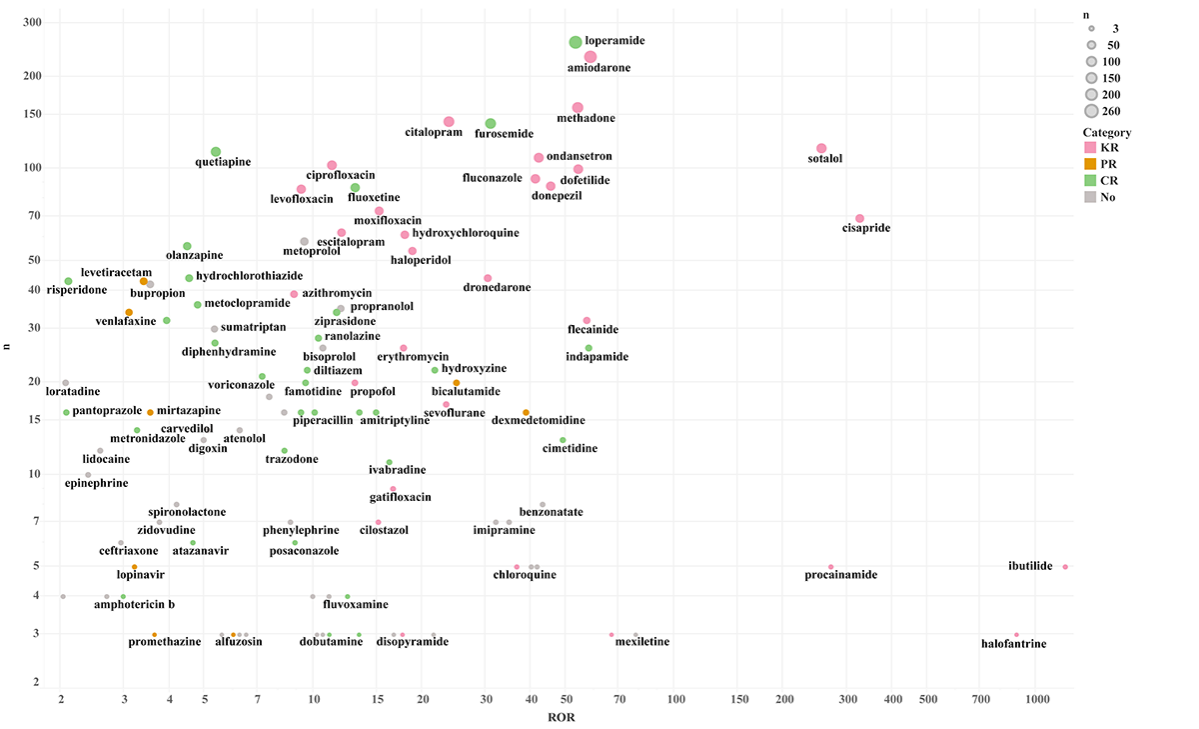
Scatterplot of the number of reports and reporting odds ratio (ROR) for torsades de pointes–related primary suspect drugs. CR: conditional risk; KR: known risk; PR: possible risk.

Of the 4313 analyzed TdP cases, 721 drugs, including 136 QT-prolonging drugs, were implicated in 3 or more cases. The most reported drugs were antiarrhythmics, antidepressants, antibacterials, antipsychotics, analgesics, antineoplastics, and others (Figure 2). Amiodarone had the most reports (n=524), followed by furosemide (n=448), methadone (n=312), loperamide (n=301), and citalopram (n=299). However, loperamide (n=260; ROR 53.19, 95% CI 46.87-60.36) was the most frequently reported primary suspect drug, followed by amiodarone (n=233; ROR 58.48, 95% CI 51.18-66.82), citalopram (n=143; ROR 23.72, 95% CI 20.06-28.04), methadone (n=159; ROR 53.91, 95% CI 45.95-63.26), furosemide (n=141; ROR 30.93, 95% CI 26.13-36.62), and sotalol (n=117; ROR 255.06, 95% CI 210.53-309.02). Ibutilide (n=5; ROR 1206.58, 95% CI 419.04-3474.16) had the strongest signal for TdP, followed by halofantrine (n=3; ROR 884.41, 95% CI 239.35-3267.99), cisapride (n=69; ROR 325.59, 95% CI 253.16-418.74), procainamide (n=5; ROR 270.86, 95% CI 107.87-680.12), and sotalol (n=117; ROR 255.06, 95% CI 210.53-309.02), and these drugs had a known TdP risk (Figure 3).

### DDI Signals of Statistical Models

We evaluated the reports of TdP for concomitant use of 2 of the 721 drugs, totaling 8264 drug combinations, of which 4230 combinations were reported for at least 3 cases. The additive model detected the most signals with 3296 (77.9%) pairs, followed by the combination risk ratio model with 2574 (60.8%) pairs, the chi-square statistic model with 2243 (53%) pairs, and the Ω shrinkage measure model with 2236 (52.9%) pairs.

### Commonality of DDI Signals Detected

Table 3 shows the κ coefficients and proportionate agreements for *P_positive_* and *P_negative_* among the 4 frequency statistical models. The chi-square statistics showed the greatest similarity to the Ω shrinkage measure, with κ, *P_positive_*, and *P_negative_* values of 0.972 (95% CI 0.942-1.002), 0.987, and 0.985, respectively. In contrast, the κ, *P_positive_*, and *P_negative_* values for the Ω shrinkage measure and additive models were 0.482 (95% CI 0.451-0.513), 0.808, and 0.638, respectively, with moderate similarity.

**Table 3:** The Cohen kappa coefficient (κ) and proportionate agreements for positive rating (Ppositive) and negative rating (Pnegative) among 4 frequency statistical models

Models	*P*_*positive*_	*P*_*negative*_	κ (95% CI)
Ω shrinkage measure model vs chi-square statistic model	0.987	0.985	0.972 (0.942-1.002)
Ω shrinkage measure model vs combination risk ratio model	0.904	0.874	0.780 (0.749-0.810)
Ω shrinkage measure model vs additive model	0.808	0.638	0.482 (0.451-0.513)
Chi-square statistic model vs combination risk ratio model	0.910	0.881	0.793 (0.762-0.823)
Chi-square statistic model vs additive model	0.810	0.640	0.485 (0.454-0.516)
Combination risk ratio model vs additive model	0.877	0.721	0.612 (0.578-0.646)

### DDI Signal Verification

**Figure 4:**
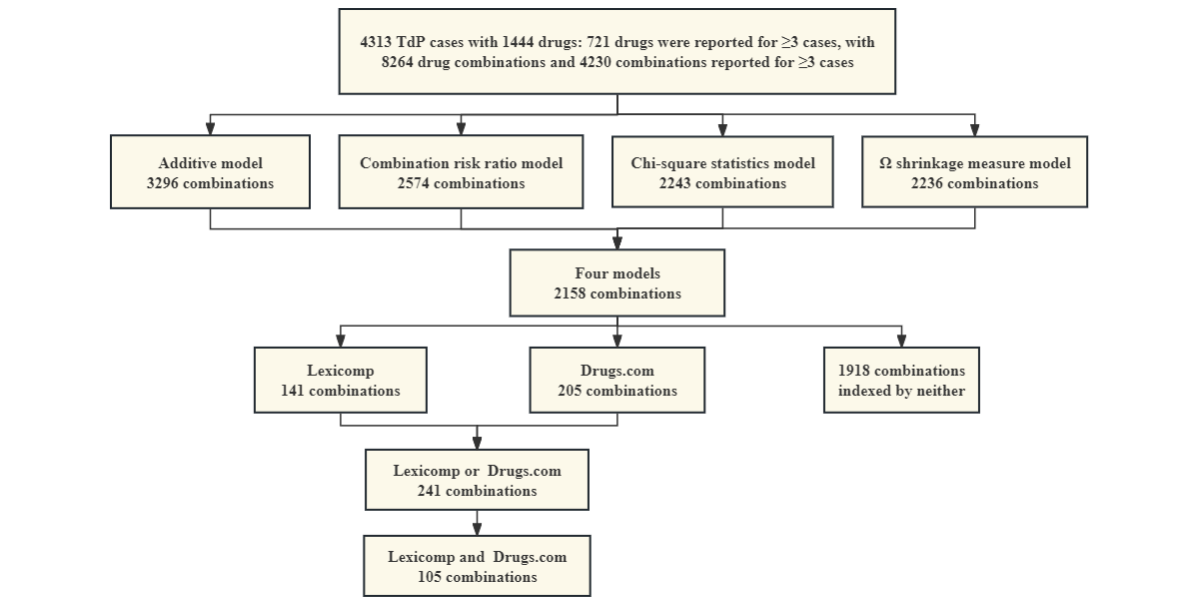
Process of drug-drug interaction signal monitoring and verification. TdP: torsades de pointes.

**Figure 5:**
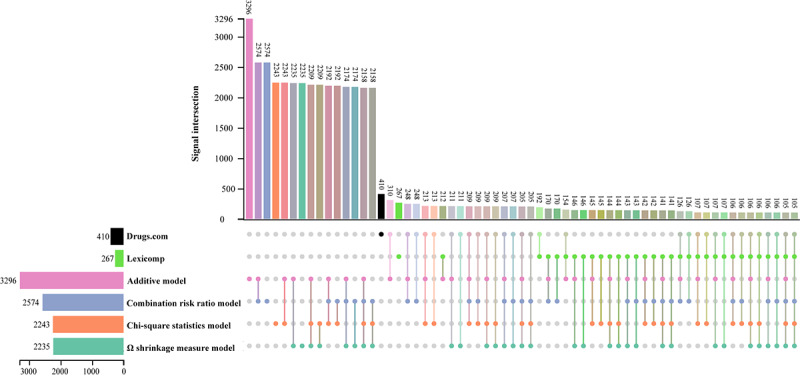
Number of drug-drug interaction signal intersections.

All 4 statistical models detected 2158 combinations (96.2% of the Ω shrinkage measure model), with 141 combinations indexed by Lexicomp, 205 combinations indexed by Drugs.com, 241 combinations indexed by either, and 1918 combinations indexed by neither as of October 8, 2023. In addition, 105 combinations were indexed by both, with 52 combinations being present in 10 or more cases (Figures 4 and 5).

### DDI Assessment

Of 2158 combinations, 409 individual drugs were involved. Categorization in CredibleMeds showed 35 “known risk,” 31 “possible risk,” and 45 “conditional risk” drugs. In Lexicomp, 12 were “high risk,” 29 were “moderate risk,” and 29 were “low risk.” The drug with the highest number of interactions was amiodarone, which interacted with 68 other drugs, followed by methadone (n=51), fluoxetine (n=46), bisoprolol (n=42), ciprofloxacin (n=41), citalopram (n=37), and lorazepam (n=37).

**Figure 6:**
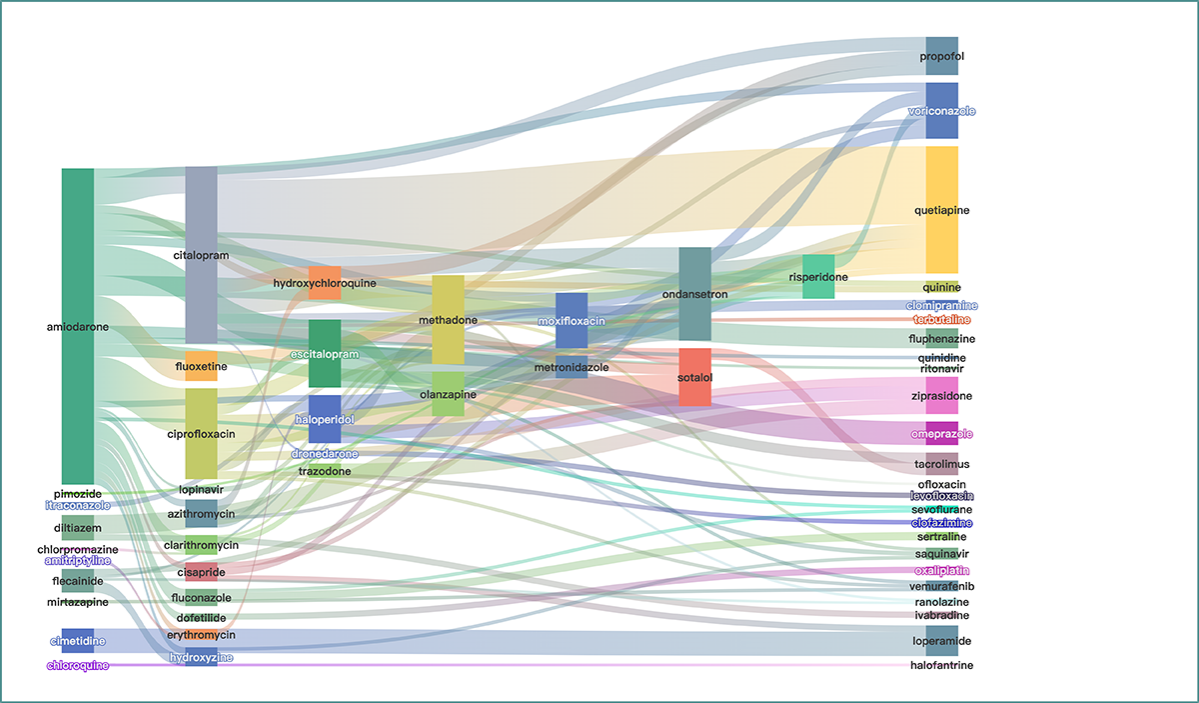
Drug-drug interactions (n=105) for torsades de pointes (detected by 4 models and indexed by Drugs.com and Lexicomp，n111≥3).

**Figure 7:**
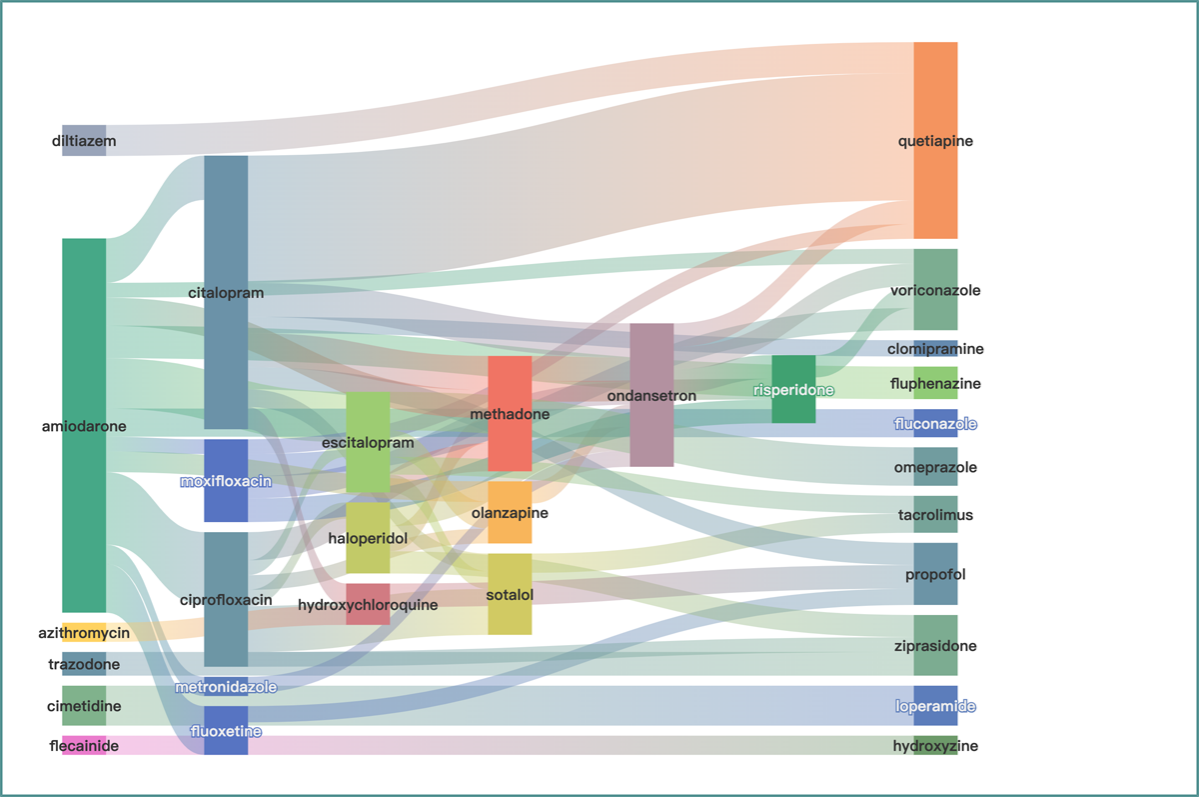
Drug-drug interactions (n=52) for torsades de pointes (detected by 4 models and indexed by Drugs.com and Lexicomp, n111≥10).

However, the drugs with the most reported interaction cases were amiodarone, citalopram, quetiapine, ondansetron, ciprofloxacin, methadone, escitalopram, sotalol, and voriconazole. The drug combinations are shown in Figures 6 and 7, with citalopram and quetiapine (n_111_=86) having the highest number of cases, followed by amiodarone and ciprofloxacin (n_111_=49), amiodarone and escitalopram (n_111_=34), amiodarone and fluoxetine (n_111_=33), ciprofloxacin and sotalol (n_111_=31), and amiodarone and citalopram (n_111_=30). The size of the nodes is proportional to the number of specific drugs with TdP, while the width of the extension branch is proportional to the number of specific combinations with TdP.

Multimedia Appendix 2 presents the characteristics of 105 drug combinations. Lexicomp classified 20 combinations as “X,” 25 as “D,” 42 as “C,” and 18 as “B.” In contrast, Drugs.com categorized 27 combinations as “Moderate risk” and 78 as “Major risk.” Additionally, 25 combinations were recorded in the literature-derived database of drug-related TdPs with enhanced clinical relevance.

**Figure 8:**
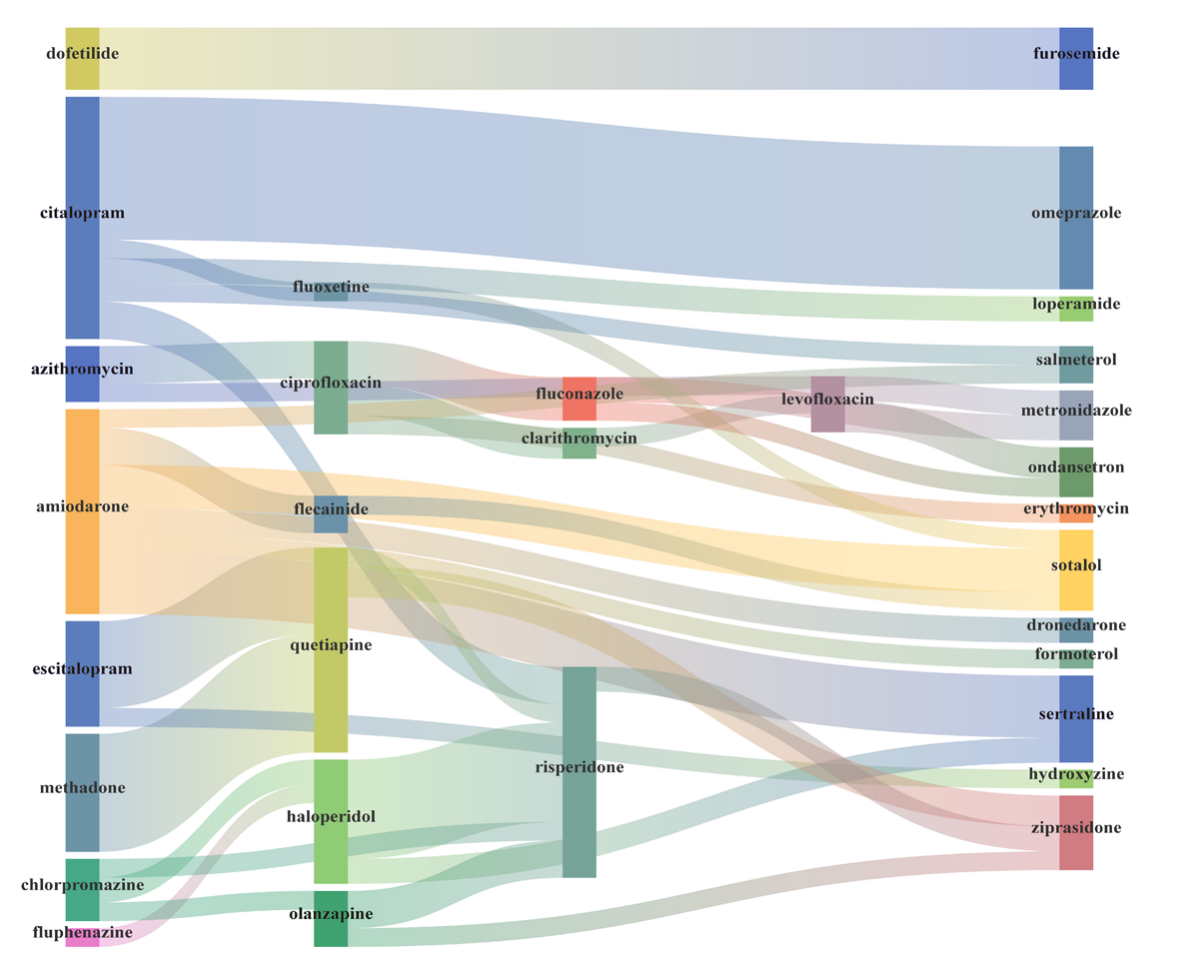
Drug-drug interactions (n=38) for torsades de pointes (indexed by Drugs.com and Lexicomp, n111≥3, but not detected by any of the 4 models).

Regrettably, 38 drug combinations indexed in both databases were not detected by any of the 4 models. These included 2 “X” class (methadone and quetiapine, quetiapine and ziprasidone), 11 “D” class, 14 “C” class, and 9 “B” class drug combinations, with amiodarone and sotalol, chlorpromazine and haloperidol, and amiodarone and dronedarone being the high-risk QT-prolonging drug combinations (Multimedia Appendix 2; Figure 8).

**Figure 9:**
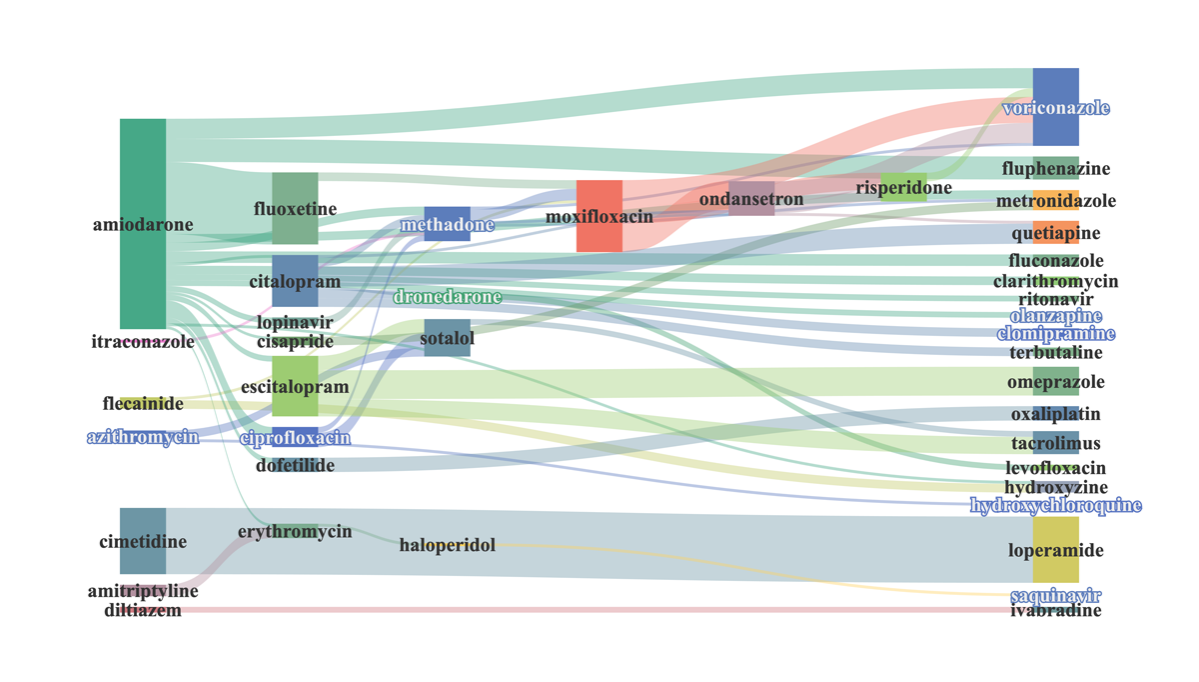
Drug-drug interactions (n=55) for torsades de pointes (drugs classified as “interacting drugs” in the Food and Drug Administration Adverse Event Reporting System, detected by the 4 models, and indexed by Drugs.com and Lexicomp).

Among the 4313 cases analyzed, 513 involved drug interaction events, with 984 interacting drugs reported for 460 cases. The most commonly interacting drugs were amiodarone, citalopram, fluoxetine, furosemide, escitalopram, ondansetron, omeprazole, moxifloxacin, and metoprolol. By matching the results of signal detection and validation of drug combinations, a total of 189 drug combinations were identified by the 4 models. Of these, 65 combinations were indexed by Lexicomp, 74 were indexed by Drugs.com, 84 were indexed by either, and 55 were indexed by both. Figure 9 illustrates the 55 DDIs classified as “interacting drugs” in FAERS that were detected by the 4 models and indexed by Drugs.com and Lexicomp. These include combinations like amiodarone and fluoxetine (n=25), loperamide and cimetidine (n=23), escitalopram and omeprazole (n=10), and moxifloxacin and ondansetron (n=10). Some combinations, although reported more frequently, were excluded from the analysis as they were either not detected by the model or not indexed in the database. For example, venlafaxine and quetiapine (n=17) and furosemide and metoprolol (n=17) were only detected by the additive model. Additionally, combinations like fluoxetine and fluphenazine (n=16) and fluconazole and ritonavir (n=13) were not indexed either Drugs.com or Lexicomp.

### Sensitivity Analyses

Despite the relatively low proportion of reports from nonprofessionals, we conducted an analysis of TdP cases reported by nonprofessionals, such as consumers and lawyers. We evaluated TdP by nonprofessionals for concomitant use of 2 of the drugs, totaling 1862 drug combinations, with 89 combinations reported for at least 3 cases. Among these, 49 combinations were detected by all 4 statistical models, and only 5 combinations were indexed by Lexicomp and Drugs.com when considering all data, including reports from nonprofessionals. Sensitivity analyses revealed that the inclusion of nonprofessional reports did not alter the final determination of the signal detection (Multimedia Appendix 2).

## Discussion

### Main Findings

This study presents the initial comparative safety evaluation of FAERS data aimed at assessing clinically significant DDIs triggering fatal TdP. Clinical evidence regarding DDIs remains limited, and not all combinations of QTc-prolonging drugs lead to TdP, even when high-risk drugs or those with established TdP risks are involved. This study offers a comprehensive real-world analysis of drug-induced TdP, identifying both clinically significant and nonsignificant DDIs. These findings contribute valuable insights into the safety profiles of various drugs, aiding in the optimization of clinical practice.

Matsuo and Yamaori [20] attempted to use the Japanese Adverse Drug Event Report database to identify and summarize potential DDIs associated with an increased risk of drug-induced long QT syndrome. However, their methodology relied on the ROR, which is typically used for assessing safety signals of individual drugs that may not be appropriate for detecting safety signals of DDIs, potentially leading to false positive signals. To address this limitation, 4 frequency statistical models—namely, the Ω shrinkage measure, combination risk ratio, chi-square statistic, and additive model—were used to detect DDI signals in our study, thereby greatly enhancing the validity and comprehensiveness of the identified signals. Furthermore, the verification of these signals was conducted using the Drugs.com, Lexicomp, and literature databases, thereby ensuring the credibility and reliability of the DDI data.

Consistent with previous studies, the Ω shrinkage measure model demonstrated the most conservative signal detection, while the chi-square statistic model exhibited the closest similarity in signal detection tendency to the Ω shrinkage measure model [16]. In contrast, the additive model detected considerably more signals [5] and had less similarity in signal detection tendency with the other models; therefore, care must be taken when interpreting the signals. For a specific positive signal result, the more models that detected it, the more reliable the result is. In this study, 2158 combinations were detected by the 4 frequency statistical models, of which 241 combinations were indexed by Drugs.com or Lexicomp and 105 were indexed by both. In addition, 171 combinations were indexed by only 1 of the databases, which was related to the poor consistency between the 2 databases [11] due to the various rating criteria and procedures and definitions of different levels of acceptable risk. Drugs.com validated more interactions than Lexicomp, which may be related to the fact that Drugs.com can recognize brand names. Despite using an open-access database of literature-derived, drug-related TdP cases containing 624 TdP cases from 424 papers to evaluate the clinical relevance of DDIs, only concomitant drugs were documented in all cases except for suspected drugs, with no recorded interacting drugs [9]. Unsurprisingly, limited clinical evidence on DDIs is available, owing to the inherent difficulty of judging when interactions lead to AEs. However, DDIs do exist, which highlights the value of detecting clinically significant DDIs based on real-world data.

In our study, a list containing all potential DDIs related to TdP from FAERS was provided. To the best of our knowledge, this is the most comprehensive list derived from a pharmacovigilance database so far. Consistent with the results of existing studies, antiarrhythmics, antidepressants, antipsychotics, antibacterials, analgesics, and antineoplastics drugs, such as amiodarone, citalopram, quetiapine, ondansetron, ciprofloxacin, methadone, escitalopram, sotalol, and voriconazole, were the most common interacting drugs causing TdP [20,21]. Additionally, diuretics were also common interacting drugs causing TdP, and the potential mechanism underlying this may be related to electrolyte disorder caused by diuretics. The most common combinations were citalopram and quetiapine, amiodarone and ciprofloxacin, amiodarone and escitalopram, amiodarone and fluoxetine, ciprofloxacin and sotalol, and amiodarone and citalopram, and previous studies have proven these DDIs are closely related to TdP [22-24]. Trazodone may lead to QTc prolongation and TdP, which is potentially fatal even without risk factors for QTc prolongation [25]. In addition, co-interaction with ziprasidone increases the risk of TdP. Therefore, we can use this report’s findings to quickly understand the DDIs that commonly result in TdP in the real world. However, it is worth noting that a high reporting ratio does not always represent a high risk because, for different combinations, the frequency of drug use will vary greatly, which will directly affect the proportion of TdP reports.

The development of TdP in the context of DDIs is likely attributable to two key mechanisms: altered drug concentrations due to metabolic inhibition or induction and superimposed pharmacological effects on cardiac ion channels. For instance, co-administration of drugs that prolong the QT interval may synergistically increase the risk of TdP. Moreover, inhibition of the metabolism of a QT-prolonging drug by a co-administered agent could lead to elevated plasma concentrations, thereby exacerbating the risk. Amiodarone is the most widely interacting drug, with interactions with other 68 drugs, followed by methadone, fluoxetine, bisoprolol, ciprofloxacin, and citalopram [26-28], which interact with 51, 46, 42, 41, and 37 other drugs, respectively. Therefore, additional caution and increased electrocardiogram monitoring may be still warranted when these drugs are combined, especially for patients with risk factors such as increasing age, female sex, bradycardia, heart failure, history of ventricular arrhythmias, use of diuretics, hypothyroidism, hypokalemia, hypomagnesemia, and hypocalcemia.

In addition to offering a complete list of drugs, our study provides novel perspectives and opportunities for further exploration. As real-world negative reporting is missing, counterexamples that could serve as contradictory evidence may exist. Therefore, identifying negative DDI pairs shall enhance the confidence of health care professionals with a level of certainty on DDIs, which in turn will improve medical research and decision-making. It is widely accepted that co-administration of QTc-prolonging drugs, especially those with a known risk or high risk for TdP, heighten the risk of TdP. Our research identified 38 DDIs for QTc prolongation indexed by Drugs.com and Lexicomp but were not detected by any of the 4 models. This finding supports the notion that not all combinations of QTc-prolonging drugs result in TdP, even when involving high-risk drugs or those with a known risk of TdP. There is only a limited increase in QTc prolongation with concurrent use of QTc-prolonging drugs, and that magnitude of TdP risk depends on the specific drugs involved and patient risk factors. If clinical decision support systems generate alerts for QTc DDI whenever 2 QTc-prolonging drugs with a known risk of TdP are combined, irrespective of the clinical relevance of these interactions, a substantial number of false-positive alerts may be produced. The rising incidence of false-positive QTc DDI alerts can contribute to alert fatigue among physicians, potentially leading to the disregard of important warnings. Furthermore, these false-positive alerts may prompt unwarranted discontinuation or substitution of medications, thereby compromising the efficacy of clinical treatments. Consequently, identifying negative QTc DDIs is a rational approach to mitigating the challenge of alert fatigue. Nevertheless, caution must be exercised even when a signal is identified as negative, due to the inherent limitations of the FAERS database.

We acknowledge the inherent limitations of our study. First, our analysis primarily focused on the potential for TdP resulting from the concomitant use of 2 drugs. However, in patients with polypharmacy, there are documented cases of concurrent use of 3 or more suspect drugs. This raises the possibility that a third drug may act as a confounding factor in the relationship between coadministration and TdP risk, which was not accounted for in our analysis. Second, the use of a spontaneous reporting system inherently introduces various biases into the signal detection process, thus rendering the obtained signals as hypotheses rather than definitive conclusions. Moreover, the absence of a universally accepted gold standard for DDI signal detection further impacts the reliability of our findings. Although we devoted significant attention to the interpretation of results in the context of signal research for DDIs, validation efforts have been conducted using resources such as Lexicomp and Drugs.com. Unfortunately, there is a paucity of reliable information on DDIs, and the available data exhibit substantial variability across different drug information resources. Furthermore, there is a scarcity of robust clinical evidence on DDIs. Consequently, it is challenging to use “real” true data for signal verification. Third, although 2 databases were used for signal validation, there exists a risk of overlooking genuine DDIs due to inconsistencies between the databases; when considering only the DDIs present in both as true signals, some valid associations may have been excluded. Fourth, the failure to convert drug trade names to their generic counterparts prior to analysis may have resulted in an elevated number of DDI-positive signals. Nevertheless, this did not substantially affect the outcomes, as the analysis exclusively included DDIs validated by Lexicomp and Drugs.com using generic names. Fifth, we did not initially stratify the analysis based on the reporter population, as reports from nonprofessionals, such as lawyers and consumers, made up only 6.7% of the total. However, despite this small proportion, we could not completely rule out the potential influence of these nonprofessional reports on the signal detection results due to their limited ability to differentiate between AE symptoms and underlying disease symptoms. Last, the FAERS database lacks sufficient clinical details to exclude comorbidities, such as heart-related diseases, as potential contributing factors to TdP cases. Future research should integrate more comprehensive clinical data or conduct prospective studies to better distinguish between medication-induced and disease-induced TdP, thereby enhancing the reliability of such analyses.

### Conclusion

This study provides a preliminary overview of potential culprit DDIs, as well as negative DDIs, for TdP in real-world settings. These findings offer valuable insights for regulators, health care professionals, and stakeholders involved in DDI management. However, it is important to acknowledge the limitations inherent to our study stemming from the reliance on a pharmacovigilance database. It is crucial to recognize that DDI signals only indicate a statistical association between drug combinations and AEs and should not be mistaken for confirmed causal relationships. Further validation through well-designed clinical studies is necessary to establish a causal relationship. Although we validated signals using DDI resources and literature-based evidence, the available clinical evidence is limited and subject to significant inconsistencies. Therefore, in clinical practice, DDI signals should be considered as supplementary evidence rather than a substitute for the expertise of cardiologists and clinical pharmacists.

Although our study provides promising initial findings regarding the identification of high-priority DDIs for TdP, the scalability of these findings should be framed as hypotheses for future research. Specifically, the ability to generalize these results for clinical settings requires further investigation. Future studies should integrate clinical data from electronic health records or prospective trials to validate our findings and provide more actionable insights for clinicians.

## Appendix

Multimedia Appendix 1 Detection of clinically significant drug-drug interactions in fatal torasdes de pointes.

Multimedia Appendix 2 Characteristics of drug-drug interactions for torsades des pointes.

## Abbreviations

AE: adverse event
DDI: drug-drug interaction.
FAERS: Food and Drug Administration Adverse Event Reporting System
MedDRA: Medical Dictionary for Regulatory Activities
PT: preferred term
QTc: heart rate–corrected QT interval
ROR: reporting odds ratio
TdP: torsades de pointes
