# Supplementary material for: Detection of Clinically Significant Drug-Drug Interactions in Fatal Torsades de Pointes: Disproportionality Analysis of the Food and Drug Administration Adverse Event Reporting System
Source: J Med Internet Res. 2025 Mar 25;27:e65872. doi: 10.2196/65872 (PMC11979527; doi:10.2196/65872)
Supplement: Multimedia Appendix 1 [file jmir_v27i1e65872_app1.docx]

**Appendix**

**Table 1.** The four-by-two contingency table for signal detection of DDI

|  | Target AE^a^ | Other AEs | Total |
| --- | --- | --- | --- |
| Concomitant use of *drug D*_1_ and *drug D*_2_ | *n*_111_^b^ | *n*_110_ | *n*_11+_ |
| *drug D*_1_ without *drug D*_2_ | *n*_101_ | *n*_100_ | *n*_10+_ |
| *drug D*_2_ without *drug D*_1_ | *n*_011_ | *n*_010_ | *n*_01+_ |
| Neither *drug D*_1_ nor *drug D*_2_ | *n*_001_ | *n*_000_ | *n*_00+_ |
| Total | *n*_++1_ | *n*_++0_ | *n*_+++_ |

^a^ AE: adverse event.

*^b^ n*: the number of reports.

Targeted AE rates for different drug exposure scenarios were as follows:

$f_{00}=\frac{n_{001}}{n_{00+}}, f_{10}=\frac{n_{101}}{n_{10+}}, f_{01}=\frac{n_{011}}{n_{01+}}, f_{11}=\frac{n_{111}}{n_{11+}}$.

**Table 2.** The two-by-two contingency table for signal detection of DDIs

|  | *drug D*_2_ | not *drug D2* |
| --- | --- | --- |
| *drug D*_1_ | $p_{11}={n_{111}}/{n_{11+}}$ | $p_{10}={n_{101}}/{n_{10+}}$ |
| not *drug D*_1_ | $p_{01}={n_{011}}/{n_{01+}}$ | $p_{00}={n_{001}}/{n_{00+}}$ |

**Ω Shrinkage Measure Model**

The Ω shrinkage measure is based on the 4 × 2 contingency table, and the signal is obtained by calculating the logarithm of an observed to expected ratio.

$$\Omega={log}_{2}\frac{n_{111}+0.5}{E_{111}+0.5}$$

$$g_{11}=1-\frac{1}{\max\left( \frac{f_{00}}{1-f_{00}}, \frac{f_{10}}{1-f_{10}} \right)+ \max\left( \frac{f_{00}}{1-f_{00}}, \frac{f_{01}}{1-f_{01}} \right)- \frac{f_{00}}{1-f_{00}} +1}$$

When *f*_10_ < *f*_00_ (which denote no risk of AE caused by *drug D*_1_), the most sensible estimator *g*_11_ = max (*f*_00_, *f*_01_) is yielded and the *vice versa* when *f*_01_ < *f*_00_.

$$E_{111}=g_{11}\times n_{11+}$$

$$\mathrm{Var}\left( \Omega_{0} \right)=Var\left( \log_{2}\frac{n_{111}}{E_{111}} \right)\approx\frac{1}{n_{111}{\log\left( 2 \right)}^{2}}$$

Where, *n*_111_ is the number of reports and *E*_111_ is the expected value.

$$\Omega_{025}=\Omega-\frac{\phi\left( 0.975 \right)}{\log\left( 2 \right)\sqrt{n_{111}}}$$

Where, *ϕ* (0.975) is 97.5% of the standard normal distribution. When Ω_025_ > 0, it is considered a positive signal.

**Chi-Square Statistics Model**

The chi-square statistics proposed by Gosho et al. to detect the signal of potential DDIs. Gosho et al. prepared the following measure “χ” to estimate the discrepancy between the observed and expected number of events with a specific drug (Table 1).

$$\chi=\frac{n_{111}-E_{111}-0.5}{\sqrt{E_{111}}}$$

Where, *n*_111_ is the number of reports, *E*_111_ is the expected value, which is the same as the estimate of the expected number of reports for the Ω shrinkage measure. Ultimately, χ > 2 is considered a signal of DDIs.

**Combination Risk Ratio Model**

When *n*_111_ ≥ 3, $\text{PRR}_{\text{drug D1 ∩ drug D2}}$ > 2, χ^2^*_drug D_*_1 ∩_ *_drug D_*_2_ > 4, *CRR*> 2, it was a signal of DDIs.

$$\text{Combination risk ratio (CRR)=}\frac{\text{PRR}_{\text{drug D1 ∩ drug D2}}}{\text{max (}\text{PRR}_{\text{drug D1}}\text{,}\text{ PRR}_{\text{drug D2}}\text{)}}\text{ }$$

$$\text{PRR=}\frac{\left( {N_{11}}/{N_{1+}} \right)}{\left( {N_{01}}/{N_{0+}} \right)}$$

$$\chi^{2}=\frac{n_{+++}\times\left( \left| N_{11} \right.\times N_{00}-N_{10}\times\left. N_{01} \right|-{n_{+++}}/2 \right)^{2}}{N_{1+}\times N_{+1}\times N_{0+}\times N_{+0}}$$

In particular, to calculate the PRR and the $\chi^{2}$ of $drug D1 \bigcap drug D2$, drug D1 and drug D2, replace them as follows:

${drug D1\cap drug D2: N}_{11}=n_{111},N_{00}=n_{000}+n_{010}+n_{100},N_{10}=n_{110},N_{01}=n_{001}+n_{011}+n_{101},N_{1+}=n_{11+},N_{+1}=n_{++1},N_{0+}=n_{00+}+n_{01+}+n_{10+},N_{+0}=n_{++0}$.

$\text{drug D1: }N_{11}=n_{111}+n_{101},N_{00}=n_{000}+n_{010},N_{10}=n_{110} {+ n}_{100},N_{01}=n_{001}+n_{011},N_{1+}=n_{11+}+n_{10+},N_{+1}=n_{++1},N_{0+}=n_{00+}+n_{01+},N_{+0}=n_{++0}$.

${drug D2: N}_{11}=n_{111}+n_{011},N_{00}=n_{000}+n_{100},N_{10}=n_{110+}n_{010},N_{01}=n_{001}+n_{101},N_{1+}=n_{11+}+n_{01+},N_{+1}=n_{++1},N_{0+}=n_{00+}+n_{10+},N_{+0}=n_{++0}$.

**Additive Model**

Under the additive assumption, no interaction is established when the excess risk associated with drug D1 in the absence of drug D2 is equal to the excess risk associated with drug D1 in the presence of drug D2 (Table 2).

（*P_11_* - *P_00_*）=（*P_10_* - *P_00_*）+（*P_01_* - *P_00_*）

When *P_11_* – *P_10_* –*P_01_* + *P_00_* >0, the signals of potential DDI were detected.

**Evaluation of Commonality of Signals Detected**

The study assessed the similarity of the signals identified by each statistical model using Cohen's kappa coefficient (κ), proportionate agreement for positive rating (*P_positive_*) and proportionate agreement for negative rating (*P_negative_*). The κ provides a measure that adjusts the observed agreement (Po) for the chance agreement (Pe) and is defined as:

$$Cohen' kappa coeficient (\kappa)=\frac{p_{0}-p_{e}}{1-p_{e}}$$

95% confidence interval (CI) of kappa coefficient

*
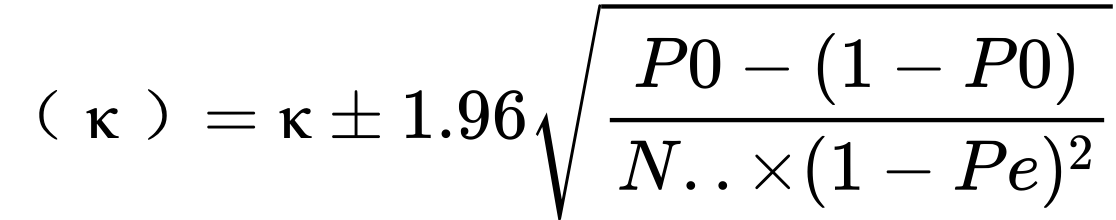
*

where *Po* and *Pe* are defined using symbols in Table 3. as:

**Table 3.** Agreement between the criterion A and the criterion B

|  | | Criterion B | |  |
| --- | --- | --- | --- | --- |
|  |  | *Yes* | *No* | Total |
| Criterion A | *Yes* | *N_yy_* | *N_yn_* | *N_y._* |
|  | *No* | *N_ny_* | *N_nn_* | *N_n._* |
|  | Total | *N_.y_* | *N_.n_* | *N_.._* |

$$P_{o}=\frac{N_{\mathrm{yy}}+N_{\mathrm{nn}}}{N_{..}}$$

$$P_{e}=\frac{N_{y.}}{N_{..}}\times\frac{N_{.y}}{N_{..}}+\frac{N_{n.}}{N_{..}}\times\frac{N_{.n}}{N_{..}}$$

The Cohen's kappa coefficient, which ranges from -1 to 1, indicates the level of agreement between observed and expected values. A perfect observed agreement (*P_o_* = 1) corresponds to a kappa value of 1, while an observed agreement equal to the expected agreement (*P_o_* = *P_e_*) results in a kappa value of 0. A scenario where there is no agreement between positive and negative values (*N_yy_* = *N_nn_* = 0 and *N_yn_* = *N_ny_* = *N/2*) leads to a kappa value of -1.

The other 2 measures *P_positive_* and *P_negative_* are defined, respectively, as:

$$P_{\mathrm{positive}}=\frac{{2N}_{\mathrm{yy}}}{N_{y.}+N_{.y}}$$

$$P_{\mathrm{negative}}=\frac{{2N}_{\mathrm{nn}}}{N_{n.}+N_{.n}}$$

**Table 4.** The severity classification of DDIs in Lexicomp® and Drugs.com^®^

| Lexicomp^®^ | Drugs.com^®^ |
| --- | --- |
| X: Avoid combination  Clinically significant and generally considered contraindicated; the risk of DDI outweighs the benefit | Major  Highly clinically significant and avoid combinations; the risk of DDI outweighs the benefit |
| D: Consider therapy modification  Clinically significant and aggressive monitoring, empiric dosage changes, or alternative agents; patient-specific assessment wether benefit outweighs risk | Moderate  Moderately clinically significant and usually avoid combinations; use only under special circumstances |
| C: Monitor therapy  Clinically significant but benefit usually outweighs risk, dosage adjustment may be needed | Minor  Minimally clinically significant; minimise risk; assess risk and consider an alternative drug, take steps to circumvent the interaction risk and/or institute a monitoring plan |
| B: No action needed  little to No evidence of clinical concern  A: No known interaction  neither PK Nor PD DDI is demonstrated | Unknown  No DDI information available |
