# Supplementary material for: Detection of Clinically Significant Drug-Drug Interactions in Fatal Torsades de Pointes: Disproportionality Analysis of the Food and Drug Administration Adverse Event Reporting System
Source: J Med Internet Res. 2025 Mar 25;27:e65872. doi: 10.2196/65872 (PMC11979527; doi:10.2196/65872)
Supplement: Multimedia Appendix 2 [file jmir_v27i1e65872_app2.docx]

**Supplementary Material.**

**Table 1.** Characteristics of 105 DDIs for Torsades des Pointes (detected by four models and indexed by Drugs.com® and Lexicomp®, n111≥3)

| Drug 1 (ROR, N) | Drug 1 Risk Category | | Drug 2 (ROR, N) | Drug 1 Risk Category | | Combination Risk Category | | Literature Database | Cases  (n_111_) | Ω_025_^a^ | χ^b^ | CRR^c^ (PRR^d^, χ^2e^) | Additive model |
| --- | --- | --- | --- | --- | --- | --- | --- | --- | --- | --- | --- | --- | --- |
|  | Lexicomp^®^ | CredibleMeds^®^ |  | Lexicomp^®^ | CredibleMeds^®^ | Lexicomp^®^ | Drugs.com^®^ |  |  |  |  |  |  |
| Citalopram (13.24, 299) | M^f^ | KR^g^ | Quetiapine (5.88, 231) | H^h^ | CR^i^ | X^j^ | Major | Yes^k^ | 86 | 1.58 | 13.01 | 3.54（46.89, 3741.53) | 0.01 |
| Amiodarone (48.53, 524) | H | KR | Ciprofloxacin (11.45, 200) | I^l^ | KR | C^m^ | Major | Yes | 49 | 2.32 | 15.66 | 6.78 (324.58, 15314.61) | 0.10 |
| Amiodarone (48.53, 524) | H | KR | Escitalopram (6.36, 146) | M | KR | D^n^ | Major | Yes | 34 | 1.49 | 8.73 | 3.78 (180.34, 5841.69) | 0.05 |
| Amiodarone (48.53, 524) | H | KR | Fluoxetine (10.42, 234) | L^o^ | CR | C | Major | Yes | 33 | 1.89 | 10.88 | 5.53 (264.87, 8351.69) | 0.08 |
| Ciprofloxacin (11.45, 200) | I | KR | Sotalol (76.65, 221) | H | KR | C | Major | Yes | 31 | 2.58 | 15.16 | 9.02（673.32, 20006.85) | 0.23 |
| Amiodarone (48.53, 524) | H | KR | Citalopram (13.24, 299) | M | KR | X | Major | Yes | 30 | 1.11 | 6.52 | 3.37 (160.89, 4578.14) | 0.04 |
| Cimetidine (22.17, 32) | I | CR | Loperamide (28.66, 301) | L | CR | B^p^ | Major | Yes | 27 | 3.65 | 25.27 | 25.14（713.52, 18396.12) | 0.26 |
| Escitalopram (6.36, 146) | M | KR | Omeprazole (2.11, 130) | I | CR | C | Moderate | No^q^ | 26 | 0.88 | 5.25 | 2.7（17.1, 376.13) | 0.00 |
| Citalopram (13.24, 299) | M | KR | Methadone (28.34, 312) | H | KR | X | Major | No | 23 | 0.35 | 3.09 | 2.46 (68.91, 1464.73) | 0.01 |
| Citalopram (13.24, 299) | M | KR | Ondansetron (10.40, 211) | I | KP | C | Major | No | 23 | 0.35 | 3.11 | 2.94 (38.71, 803.72) | 0.01 |
| Amiodarone (48.53, 524) | H | KR | Fluphenazine (45.24, 29) | L | NA^r^ | C | Major | No | 22 | 4.03 | 32.75 | 55.81（2665.98, 55708.44) | 0.98 |
| Diltiazem (8.33, 119) | I | CR | Quetiapine (5.88, 231) | H | CR | D | Moderate | No | 21 | 2.21 | 11.25 | 10.92（90.71, 1766.58) | 0.03 |
| Amiodarone (48.53, 524) | H | KR | Clarithromycin (12.82, 103) | M | KR | X | Major | Yes | 19 | 1.64 | 7.95 | 5.64 (269.24, 4793.58) | 0.08 |
| Ciprofloxacin (11.45, 200) | I | KR | Methadone (28.34, 312) | H | KR | C | Major | Yes | 19 | 2.24 | 11.12 | 10.55（296.01, 5273.79) | 0.10 |
| Amiodarone (48.53, 524) | H | KR | Fluconazole (16.41, 142) | M | KR | D | Major | Yes | 19 | 1.15 | 5.89 | 4.16 (198.50, 3524.43) | 0.05 |
| Amiodarone (48.53, 524) | H | KR | Methadone (28.34, 312) | H | KR | D | Major | No | 19 | 3.08 | 17.64 | 25.84 (1234.59, 22112.41) | 0.44 |
| Escitalopram (6.36, 146) | M | KR | Olanzapine (4.45, 101) | M | CR | C | Moderate | No | 18 | 0.57 | 3.71 | 3.22 (20.41, 312.02) | 0.00 |
| Haloperidol (13.96, 114) | H | KR | Methadone (28.34, 312) | H | KR | D | Major | No | 17 | 2.00 | 9.53 | 9.60 (269.45, 4267.03) | 0.09 |
| Methadone (28.34, 312) | H | KR | Ondansetron (10.40, 211) | I | KR | D | Major | No | 17 | 0.57 | 3.65 | 2.93 (82.15, 1278.30) | 0.02 |
| Trazodone (4.85, 89) | L | CR | Ziprasidone (9.94, 55) | H | CR | C | Major | No | 16 | 1.36 | 6.44 | 4.83（48.51, 695.43) | 0.01 |
| Haloperidol (13.96, 114) | H | KR | Ondansetron (10.40, 211) | I | KR | C | Major | No | 16 | 1.68 | 7.84 | 8.66（120.32, 1769.96) | 0.04 |
| Moxifloxacin (17.34, 128) | M | KR | Risperidone (3.43, 106) | M | CR | C | Major | No | 16 | 3.48 | 23.48 | 37.44（645.39, 9627.81) | 0.24 |
| Hydroxychloroquine (4.30, 101) | L | KR | Propofol (17.04, 82) | M | KR | B | Moderate | No | 16 | 2.80 | 14.78 | 15.81（267.9, 3978.39) | 0.09 |
| Ondansetron (10.40, 211) | I | KR | Risperidone (3.43, 106) | M | CR | C | Moderate | No | 16 | 2.45 | 12.09 | 12.61（130.66, 1924.62) | 0.04 |
| Ondansetron^I^ (10.40, 211) | I | KR | Quetiapine (5.88, 231) | H | CR | D | Moderate | No | 16 | 0.91 | 4.76 | 4.28 (44.37, 633.44) | 0.01 |
| Citalopram (13.24, 299) | M | KR | Propofol (17.04, 82) | M | KR | C | Moderate | No | 15 | 2.51 | 12.44 | 18.85（319.39, 4434.09) | 0.11 |
| Moxifloxacin (17.34, 128) | M | KR | Voriconazole (9.22, 47) | M | CR | C | Major | No | 15 | 2.17 | 10.23 | 11.08（191.02, 2640.38) | 0.06 |
| Moxifloxacin (17.34, 128) | M | KR | Ondansetron (10.40, 211) | I | KR | C | Major | No | 15 | 1.87 | 8.61 | 9.73（167.75, 2315.14) | 0.05 |
| Risperidone (3.43, 106) | M | CR | Voriconazole (9.22, 47) | M | CR | C | Moderate | No | 15 | 3.89 | 39.13 | 98.76（907.38, 12650.04) | 0.34 |
| Ondansetron (10.40, 211) | I | KR | Voriconazole (9.22, 47) | M | CR | C | Moderate | Yes | 15 | 1.89 | 8.72 | 10.42（107.9, 1478.95) | 0.04 |
| Citalopram (13.24, 299) | M | KR | Hydroxychloroquine (4.30, 101) | L | KR | C | Major | Yes | 15 | 1.17 | 5.61 | 4.48（59.06, 796.55) | 0.02 |
| Haloperidol (13.96, 114) | H | KR | Ziprasidone (9.94, 55) | H | CR | D | Major | No | 15 | 0.29 | 2.72 | 2.85 (39.53, 523.77) | 0.01 |
| Olanzapine (4.45, 101) | M | CR | Ondansetron (10.40, 211) | I | KR | C | Moderate | No | 15 | 0.70 | 3.98 | 3.34 (34.56, 454.48) | 0.01 |
| Amiodarone (48.53, 524) | H | KR | Olanzapine (4.45, 101) | M | CR | D | Moderate | Yes | 14 | 1.68 | 7.59 | 5.84（280.1, 3609.02) | 0.09 |
| Methadone (28.34, 312) | H | KR | Risperidone (3.43, 106) | M | CR | D | Major | No | 14 | 0.95 | 4.77 | 3.42（96.01, 1219.52) | 0.03 |
| Sotalol (76.65, 221) | H | KR | Tacrolimus (1.38, 38) | L | PR^s^ | C | Major | No | 13 | 1.99 | 9.00 | 7.85（586.19, 7002.03) | 0.20 |
| Amiodarone (48.53, 524) | H | KR | Metronidazole (3.95, 42) | L | CR | C | Moderate | Yes | 13 | 1.04 | 5.00 | 3.51（167.89, 1987.69) | 0.05 |
| Flecainide (28.01, 78) | M | KR | Hydroxyzine (6.20, 59) | L | CR | B | Moderate | Yes | 13 | 2.77 | 14.54 | 18.9（524.02, 6256.75) | 0.19 |
| Azithromycin (7.75, 78) | M | KR | Hydroxychloroquine (4.30, 101) | L | KR | C | Major | Yes | 13 | 0.17 | 2.33 | 2.47 (19.08, 204.51) | 0.00 |
| Escitalopram (6.36, 146) | M | KR | Tacrolimus (1.38, 38) | L | PR | B | Major | No | 12 | 2.60 | 13.08 | 15.76（100.05, 1077.18) | 0.04 |
| Citalopram (13.24, 299) | M | KR | Sotalol (76.65, 221) | H | KR | X | Major | No | 12 | 0.66 | 3.72 | 3.15 (234.68, 2557.30) | 0.06 |
| Escitalopram (6.36, 146) | M | KR | Sotalol (76.65, 221) | H | KR | D | Major | No | 12 | 0.79 | 4.12 | 3.22 (239.98, 2615.50) | 0.06 |
| Fluoxetine (10.42, 234) | L | CR | Propofol (17.04, 82) | M | KR | B | Moderate | No | 11 | 2.17 | 9.94 | 16.14（273.37, 2713.04) | 0.09 |
| Citalopram (13.24, 299) | M | KR | Clomipramine (9.75, 16) | M | CR | C | Major | No | 11 | 1.71 | 7.45 | 8.1（106.75, 1046.77) | 0.03 |
| Ciprofloxacin (11.45, 200) | I | KR | Haloperidol (13.96, 114) | H | KR | C | Major | No | 11 | 1.84 | 8.07 | 13.25（183.97, 1818.93) | 0.06 |
| Metronidazole (3.95, 42) | L | CR | Ondansetron (10.40, 211) | I | KR | B | Moderate | No | 11 | 0.32 | 2.71 | 2.65 (27.46, 254.10) | 0.01 |
| Amiodarone (48.53, 524) | H | KR | Voriconazole (9.22, 47) | M | CR | X | Major | No | 10 | 1.51 | 6.52 | 6.63 (316.49, 2831.86) | 0.10 |
| Ciprofloxacin (11.45, 200) | I | KR | Olanzapine (4.45, 101) | M | CR | C | Major | No | 10 | 1.49 | 6.42 | 7.66（88.32, 776.7) | 0.03 |
| Ciprofloxacin (11.45, 200) | I | KR | Ziprasidone (9.94, 55) | H | CR | C | Major | No | 10 | 2.73 | 15.27 | 43.15（492.32, 4415.63) | 0.18 |
| Moxifloxacin (17.34, 128) | M | KR | Quetiapine (5.88, 231) | H | CR | X | Major | No | 10 | 2.06 | 9.24 | 13.29（229.18, 2045.44) | 0.08 |
| Ciprofloxacin (11.45, 200) | I | KR | Escitalopram (6.36, 146) | M | KR | B | Major | No | 10 | 0.39 | 2.87 | 3.50 (39.92, 340.83) | 0.01 |
| Amiodarone (48.53, 524) | H | KR | Moxifloxacin (17.34, 128) | M | KR | X | Major | No | 10 | 1.03 | 4.73 | 5.06 (241.68, 2158.04) | 0.07 |
| Azithromycin (7.75, 78) | M | KR | Trazodone (4.85, 89) | L | CR | B | Moderate | Yes | 9 | 1.24 | 5.40 | 7.32（56.55, 436.38) | 0.02 |
| Fluconazole (16.41, 142) | M | KR | Sertraline (2.03, 78) | L | CR | B | Moderate | Yes | 9 | 0.34 | 2.72 | 2.56（42.41, 323.13) | 0.01 |
| Amiodarone (48.53, 524) | H | KR | Dofetilide (53.53, 124) | H | KR | D | Major | No | 9 | 0.39 | 2.84 | 4.51 (236.84, 1881.14) | 0.06 |
| Amiodarone (48.53, 524) | H | KR | Hydroxychloroquine (4.30, 101) | L | KR | C | Major | No | 9 | 0.18 | 2.30 | 2.14 (102.23, 802.36) | 0.02 |
| Amiodarone (48.53, 524) | H | KR | Erythromycin (10.37, 37) | M | KR | X | Major | No | 8 | 1.41 | 6.04 | 7.30 (348.50, 2431.80) | 0.11 |
| Cisapride (296.27, 82) | H | KR | Metronidazole (3.95, 42) | L | CR | C | Moderate | No | 8 | 0.72 | 3.69 | 3.46（924.28, 6474.28) | 0.26 |
| Amiodarone (48.53, 524) | H | KR | Azithromycin (7.75, 78) | M | KR | D | Major | No | 8 | 0.48 | 3.05 | 3.07 (146.61, 1014.39) | 0.04 |
| Amiodarone (48.53, 524) | H | KR | Hydroxyzine (6.20, 59) | L | CR | C | Major | Yes | 8 | 0.25 | 2.48 | 2.47 (118.10, 814.26) | 0.03 |
| Cisapride (296.27, 82) | H | KR | Loperamide (28.66, 301) | L | CR | C | Moderate | No | 7 | 0.53 | 3.17 | 3.49 (929.84, 5592.30) | 0.25 |
| Methadone (28.34, 312) | H | KR | Voriconazole (9.22, 47) | M | CR | D | Major | Yes | 7 | 1.90 | 8.75 | 16.99（476.84, 2861.53) | 0.17 |
| Diltiazem (8.33, 119) | I | CR | Ivabradine (29.96, 46) | I | CR | X | Major | Yes | 7 | 1.48 | 6.35 | 9.36（277.56, 1660.25) | 0.09 |
| Hydroxychloroquine (4.30, 101) | L | KR | Quinine (6.31, 9) | H | CR | C | Major | No | 7 | 2.68 | 25.43 | 75.71（476.84, 2861.53) | 0.18 |
| Dofetilide (53.53, 124) | H | KR | Oxaliplatin (1.84, 16) | L | KR | C | Major | No | 7 | 2.45 | 15.66 | 39.34（2066.32, 12443.2) | 0.76 |
| Clarithromycin (12.82, 103) | M | KR | Olanzapine (4.45, 101) | M | CR | C | Moderate | No | 7 | 1.95 | 9.14 | 18.45（235.4, 1406.09) | 0.08 |
| Amiodarone (48.53, 524) | H | KR | Cisapride (296.27, 82) | H | KR | D | Major | No | 7 | 1.45 | 6.22 | 8.70 (2324.61, 14000.22) | 0.77 |
| Flecainide (28.01, 78) | M | KR | Quetiapine (5.88, 231) | H | CR | D | Moderate | No | 7 | 0.79 | 3.87 | 4.69 (130.05, 771.01) | 0.04 |
| Methadone (28.34, 312) | H | KR | Moxifloxacin (17.34, 128) | M | KR | X | Major | No | 7 | 1.38 | 5.92 | 11.23 (315.20, 1887.12) | 0.10 |
| Amiodarone (48.53, 524) | H | KR | Haloperidol (13.96, 114) | H | KR | D | Major | No | 7 | 0.20 | 2.36 | 2.95 (140.88, 836.34) | 0.03 |
| Lopinavir (3.42, 17) | L | PR | Methadone (28.34, 312) | H | KR | C | Major | No | 6 | 0.77 | 3.82 | 4.65（130.63, 646.96) | 0.04 |
| Cisapride (296.27, 82) | H | KR | Ondansetron (10.40, 211) | I | KR | D | Major | No | 6 | 0.61 | 3.39 | 3.98 (1062.43, 5340.01) | 0.30 |
| Itraconazole (11.35, 25) | L | CR | Methadone (28.34, 312) | H | KR | X | Major | Yes | 6 | 2.31 | 17.09 | 70.98（1992.06, 10022.14) | 0.74 |
| Erythromycin (10.37, 37) | M | KR | Haloperidol (13.96, 114) | H | KR | C | Major | No | 6 | 1.52 | 6.80 | 16.63（230.96, 1152.29) | 0.08 |
| Dronedarone (23.16, 51) | H | KR | Levofloxacin (6.63, 116) | I | KR | X | Major | No | 6 | 1.99 | 10.57 | 26.69（612.94, 3076.13) | 0.22 |
| Amiodarone (48.53, 524) | H | KR | Quinine (6.31, 9) | H | CR | D | Major | No | 6 | 1.64 | 7.51 | 11.91（569.16, 2855.62) | 0.20 |
| Azithromycin (7.75, 78) | M | KR | Methadone (28.34, 312) | H | KR | D | Major | No | 5 | 0.76 | 3.87 | 6.48 (181.88, 727.07) | 0.06 |
| Moxifloxacin (17.34, 128) | M | KR | Sotalol (76.65, 221) | H | KR | X | Major | No | 5 | 0.65 | 3.55 | 5.74 (428.30, 1724.28) | 0.13 |
| Ciprofloxacin (11.45, 200) | I | KR | Clofazimine (8.47, 8) | M | CR | B | Moderate | No | 5 | 1.55 | 7.78 | 19.39（221.29, 886.54) | 0.08 |
| Haloperidol (13.96, 114) | H | KR | Saquinavir (8.49, 5) | M | PR | X | Major | No | 5 | 2.15 | 28.64 | 191.17 (2655.47, 10737.23) | 1.00 |
| Azithromycin (7.75, 78) | M | KR | Sotalol (76.65, 221) | H | KR | D | Major | No | 4 | 0.34 | 2.85 | 4.74 (353.98, 1076.48) | 0.10 |
| Fluoxetine (10.42, 234) | L | CR | Moxifloxacin (17.34, 128) | M | KR | B | Major | No | 4 | 0.12 | 2.32 | 5.36 (92.34, 275.69) | 0.03 |
| Methadone (28.34, 312) | H | KR | Metronidazole (3.95, 42) | L | CR | C | Moderate | Yes | 4 | 0.19 | 2.48 | 4.03（112.97, 338.82) | 0.03 |
| Mirtazapine (3.68, 59) | L | PR | Vemurafenib (2.32, 7) | M | PR | B | Major | No | 4 | 1.70 | 19.78 | 120.39（442.47, 1347.34) | 0.17 |
| Escitalopram (6.36, 146) | M | KR | Vemurafenib (2.32, 7) | M | PR | C | Major | No | 4 | 1.57 | 12.13 | 47.8（303.41, 921.7) | 0.11 |
| Hydroxyzine (6.20, 59) | L | CR | Saquinavir (8.46, 5) | M | PR | B | Major | No | 4 | 1.72 | 23.58 | 139.45（1179.93, 3604.56) | 0.44 |
| Amiodarone (48.53, 524) | H | KR | Quinidine (5.35.9) | H | KR | D | Major | Yes | 4 | 0.13 | 2.34 | 3.42（163.37, 493.09) | 0.04 |
| Methadone (28.34, 312) | H | KR | Saquinavir (8.49, 5) | M | PR | D | Major | No | 4 | 1.57 | 12.09 | 47.3（1327.42, 4056.01) | 0.49 |
| Citalopram (13.24, 299) | M | KR | Terbutaline (5.00, 5) | M | NA | C | Major | No | 4 | 1.10 | 5.83 | 12.39（163.37, 493.09) | 0.06 |
| Fluconazole (16.41, 142) | M | KR | Sevoflurane (30.22, 44) | M | KR | C | Moderate | Yes | 4 | 1.23 | 6.77 | 23.68（707.96, 2159.94) | 0.25 |
| Ciprofloxacin (11.45, 200) | I | KR | Vemurafenib (2.32, 7) | M | PR | B | Major | No | 4 | 1.56 | 11.70 | 44.32（505.69, 1540.82) | 0.19 |
| Amitriptyline (5.38, 87) | L | CR | Erythromycin (10.37, 37) | M | KR | B | Moderate | No | 4 | 0.33 | 2.83 | 5.97（61.74, 182.04) | 0.02 |
| Amiodarone (48.53, 524) | H | KR | Sevoflurane (30.22, 44) | M | KR | D | Major | No | 4 | 0.77 | 4.21 | 11.12 (530.97, 1618.21) | 0.17 |
| Citalopram (13.24, 299) | M | KR | Dronedarone (23.17, 51) | H | KR | X | Major | No | 3 | 0.24 | 2.94 | 8.95 (209.54, 431.43) | 0.07 |
| Clarithromycin (12.82 103) | M | KR | Methadone (28.34, 312) | H | KR | X | Major | No | 3 | 0.05 | 2.41 | 6.45 (180.97, 371.92) | 0.05 |
| Amiodarone (48.53, 524) | H | KR | Chlorpromazine (5.40, 11) | H | KR | D | Major | No | 3 | 0.52 | 3.98 | 9.26（442.37, 916.32) | 0.15 |
| Amiodarone (48.53, 524) | H | KR | Ritonavir (2.98, 40) | I | PR | X | Major | No | 3 | 0.03 | 2.37 | 4.39（209.54, 431.43) | 0.06 |
| Chloroquine (18.14, 13) | M | KR | Halofantrine (723.61, 3) | M | KR | C | Major | No | 3 | 1.15 | 17.15 | 3.50 (1990.68, 4140.92) | 0.75 |
| Citalopram (13.24, 299) | M | KR | Ranolazine (10.57, 41) | L | CR | B | Major | No | 3 | 0.02 | 2.32 | 7.46 (98.30, 199.77) | 0.03 |
| Flecainide (28.01, 78) | M | KR | Moxifloxacin (17.34, 128) | M | KR | C | Major | No | 3 | 0.78 | 5.62 | 26.1（723.88, 1502.61) | 0.26 |
| Amiodarone (48.53, 524) | H | KR | Lopinavir (3.42, 17) | L | PR | D | Major | No | 3 | 0.66 | 4.72 | 11.91（568.76, 1179.55) | 0.20 |
| Chlorpromazine (5.40, 11) | H | KR | Clarithromycin (12.82, 103) | M | KR | X | Moderate | No | 3 | 0.98 | 8.28 | 41.61（530.85, 1100.58) | 0.19 |
| Flecainide (28.01, 78) | M | KR | RaNolazine (10.57, 41) | L | CR | B | Moderate | No | 3 | 0.73 | 5.19 | 19.14（530.85, 1100.58) | 0.19 |
| Escitalopram (6.36, 146) | M | KR | ofloxacin (2.96, 4) | I | PR | B | Major | No | 3 | 0.96 | 7.87 | 30.6（194.21, 399.5) | 0.07 |
| Pimozide (28.80, 5) | M | KR | Risperidone (3.43, 106) | M | CR | X | Major | No | 3 | 0.11 | 2.55 | 3.04（86.55, 175.29) | 0.03 |

^a^ Ω_025,_ the signal value of the Ω Shrinkage measure model.

^b^χ, the signal value of the Chi-square statistics model.

^c^ CRR, Combination risk ratio.

^d^ PRR, proportional reporting ratio of drug D1 ∩ drug D2.

^e^χ^2^, Chi-squared of drug D1 ∩ drug D2.

^f^ M, QT-prolonging agents (Moderate Risk - Avoid) interacting drug in Lexicomp^®^ Risk Category.

^g^ KR, known risk of TdP in Lexicomp^®^ Risk Category.

^h^ H, QT-prolonging agents (Highest Risk) interacting drug in Lexicomp^®^ Risk Category.

^i^ CR, conditional of TdP in Lexicomp^®^ Risk Category.

^j^ X, Avoid combination in Lexicomp^®^ Risk Rating.

C, Monitor therapy in Lexicomp^®^ Risk Rating

^k^ Yes , presence of Drug 1 and Drug 2 combinations in the open database of drug-related TdP case literature.

^l^ I, QT-prolonging Agents (Indeterminate Risk - Avoid) interacting drug in Lexicomp^®^ Risk Category.

^m^ C, Monitor therapy in Lexicomp^®^ Risk Rating.

^n^ D, Consider therapy modification in Lexicomp^®^ Risk Rating.

^o^ L, QT-prolonging agents (Low Risk - Avoid) interacting drug in Lexicomp^®^ Risk Category.

^p^ B, No action needed in Lexicomp^®^ Risk Rating.

^q^ NO, absence of Drug 1 and Drug 2 combinations in the open database of drug-related TdP case literature.

^r^ NA, uncategorized in CredibleMed^®^.

^s^PR, possible of TdP in CredibleMeds® Risk Category.

**Table 2.** 38 DDIs for Torsades des Pointes (indexed by Drugs.com® and Lexicomp®, n_111_≥3, but not detected by any of the four models)

| Drug 1 (ROR, N) | Drug 1 Risk Category | | Drug 2 (ROR, N) | Drug 1 Risk Category | | Combination Risk Category | | Literature Database | Cases  (n_111_) | Ω_025_^a^ | χ^b^ | CRR^c^ (PRR^d^, χ^2e^) | Additive model |
| --- | --- | --- | --- | --- | --- | --- | --- | --- | --- | --- | --- | --- | --- |
|  | Lexicomp^®^ | CredibleMeds^®^ |  | Lexicomp^®^ | CredibleMeds^®^ | Lexicomp^®^ | Drugs.com^®^ |  |  |  |  |  |  |
| Citalopram (23.72, 143) | M^f^ | KR^g^ | Omeprazole (3.92, 32) | I^h^ | CR^i^ | D^j^ | Major | No^k^ | 23 | -0.90 | -1.18 | 0.81 (10.69, 191.43) | 0.00 |
| Methadone (53.91, 159) | H^l^ | KR | Quetiapine (5.37, 114) | H | CR | X^m^ | Major | Yes^n^ | 19 | -0.71 | -0.33 | 1.03 (28.87, 481.76) | 0.00 |
| Haloperidol (18.08, 54) | H | KR | Risperidone (2.10, 43) | M | CR | C^o^ | Major | No | 16 | -1.34 | -1.95 | 0.75 (10.34, 125.39) | 0.00 |
| Escitalopram (11.96, 62) | M | KR | Quetiapine (5.37, 114) | M | CR | D | Major | Yes | 14 | -1.21 | -1.38 | 1.20 (7.59, 73.48) | 0.00 |
| Dofetilide (54.10, 100) | H | KR | Furosemide (30.93, 141) | I | CR | D | Major | Yes | 10 | -1.17 | -0.81 | 0.90 (47.22, 406.57) | 0.00 |
| Amiodarone (58.48, 233) | H | KR | Sertraline (2.03, 78) | L^p^ | CR | C | Major | No | 10 | -0.97 | -0.36 | 0.85 (40.40, 345.19) | 0.00 |
| Ciprofloxacin (11.25, 103) | I | KR | Fluconazole (41.16, 93) | M | KR | B^q^ | Moderate | No | 7 | -2.30 | -2.61 | 0.65 (10.59, 51.54) | -0.01 |
| Amiodarone (58.48, 233) | H | KR | Sotalol (255.06, 117) | H | KR | D | Major | Yes | 7 | -2.50 | -3.02 | 0.52 (39.23, 223.69) | -0.03 |
| Citalopram (23.72, 143) | M | KR | Risperidone (2.10, 43) | M | CR | C | Major | No | 6 | -2.18 | -2.07 | 0.52 (6.90, 24.62) | 0.00 |
| Amiodarone (58.48, 233) | H | KR | Flecainide (57.13, 32) | M | KR | D | Major | No | 6 | -2.04 | -1.81 | 0.73 (34.64, 163.67) | -0.01 |
| Azithromycin (8.84, 39) | M | KR | Ciprofloxacin (11.25, 103) | I | KR | B | Moderate | Yes | 6 | -1.52 | -0.88 | 1.14 (12.97, 54.77) | 0.00 |
| Olanzapine (4.47, 56) | M | CR | Risperidone (2.10, 43) | M | CR | C | Moderate | No | 6 | -2.81 | -3.28 | 0.48 (2.13, 2.55) | 0.00 |
| Ciprofloxacin (11.25, 103) | I | KR | Clarithromycin (15.61, 63) | M | KR | B | Moderate | No | 5 | -1.69 | -0.94 | 1.24 (15.84, 55.44) | 0.00 |
| Levofloxacin (9.25, 86) | I | KR | Ondansetron (42.06, 109) | I | KR | C | Moderate | No | 5 | -2.24 | -1.86 | 0.71 (7.34, 21.38) | 0.00 |
| Quetiapine (5.37, 114) | M | CR | Ziprasidone (11.59, 34) | H | CR | X | Major | No | 5 | -3.02 | -3.23 | 0.44 (4.33, 9.67) | 0.00 |
| Chlorpromazine (5.40, 11) | H | KR | Haloperidol (18.80, 54) | H | KR | D | Major | No | 4 | -2.18 | -1.41 | 0.68 (9.37, 22.12) | 0.00 |
| Fluconazole (41.16, 93) | M | KR | Metronidazole (3.25, 14) | L | CR | B | Moderate | No | 4 | -2.38 | -1.70 | 0.55 (8.96, 20.88) | 0.00 |
| Amiodarone (58.48, 233) | H | KR | Dronedarone (30.38, 44) | H | KR | D | Major | No | 4 | -2.24 | -1.50 | 0.69 (32.88, 93.75) | -0.01 |
| Haloperidol (18.80, 54) | H | KR | Sertraline (2.03, 78) | L | CR | C | Major | No | 4 | -1.88 | -0.96 | 0.73 (10.07, 24.24) | 0.00 |
| Levofloxacin (9.25, 86) | I | KR | Metronidazole (3.25, 14) | L | CR | B | Moderate | No | 4 | -2.00 | -1.15 | 0.88 (5.81, 11.48) | 0.00 |
| Citalopram (23.72, 143) | M | KR | Loperamide (53.19, 260) | L | CR | B | Moderate | No | 4 | -2.56 | -1.97 | 0.56 (15.78, 41.55) | -0.01 |
| Risperidone (2.10, 43) | M | CR | Ziprasidone (11.59, 34) | H | CR | D | Major | No | 4 | -2.60 | -2.05 | 0.52 (5.16, 9.58) | 0.00 |
| Citalopram (23.72, 143) | H | KR | Fluoxetine (13.05, 87) | L | CR | D | Major | No | 3 | -3.52 | -2.86 | 0.39 (5.10, 6.21) | -0.01 |
| Amiodarone (58.48, 233) | H | KR | Formoterol | L | NA | C | Moderate | No | 3 | -2.70 | -1.69 | 0.38 (18.26, 33.20) | -0.01 |
| Azithromycin (8.84, 39) | M | KR | Levofloxacin (9.25, 86) | I | KR | C | Moderate | No | 3 | -2.58 | -1.54 | 0.80 (6.16, 8.32) | 0.00 |
| Ciprofloxacin (11.25, 103) | I | KR | Erythromycin (17.76, 26) | M | KR | B | Moderate | No | 3 | -1.90 | -0.66 | 1.37 (15.67, 27.84) | 0.00 |
| Escitalopram (11.96, 62) | M | KR | Hydroxyzine (21.68, 22) | L | CR | B | Major | No | 3 | -2.37 | -1.26 | 0.98 (6.22, 8.44) | 0.00 |
| Fluphenazine (40.10, 5) | L | NA^r^ | Haloperidol (18.80, 54) | H | KR | C | Major | No | 3 | -2.50 | -1.44 | 0.66 (29.27, 56.07) | -0.01 |
| Clarithromycin (15.61, 63) | M | KR | Levofloxacin (9.25, 86) | I | KR | C | Moderate | No | 3 | -2.74 | -1.75 | 0.59 (7.58, 11.17) | 0.00 |
| Olanzapine (4.47, 56) | M | CR | Ziprasidone (11.59, 34) | H | CR | D | Moderate | No | 3 | -3.50 | -2.83 | 0.35 (3.48, 3.11) | 0.00 |
| Amiodarone (58.48, 233) | H | KR | Salmeterol | L | NA | C | Moderate | No | 3 | -2.67 | -1.66 | 0.39 (18.60, 33.91) | -0.01 |
| Chlorpromazine (5.40, 11) | H | KR | Olanzapine (4.47, 56) | M | CR | D | Moderate | No | 3 | -1.87 | -0.63 | 1.25 (6.72, 9.45) | 0.00 |
| Flecainide (57.13, 32) | M | KR | Sotalol (255.06, 117) | H | KR | D | Major | Yes | 3 | -2.94 | -2.03 | 0.46 (34.62, 67.19) | -0.02 |
| Fluoxetine (13.05, 87) | L | CR | Sotalol (255.06, 117) | H | KR | C | Major | No | 3 | -1.61 | -0.30 | 1.06 (78.84, 159.23) | 0.00 |
| Citalopram (23.72, 143) | M | KR | Salmeterol | L | NA | B | Moderate | No | 3 | -2.37 | -1.26 | 0.52 (6.86, 9.72) | 0.00 |
| Fluconazole (41.16, 93) | M | KR | Ondansetron (42.06, 109) | I | KR | C | Moderate | No | 3 | -3.83 | -3.36 | 0.30 (4.84, 5.70) | -0.01 |
| Quetiapine (5.37, 114) | M | CR | Risperidone (2.10, 43) | M | CR | C | Moderate | No | 3 | -4.75 | -5.06 | 0.15 (0.87, 0.00) | 0.00 |
| Chlorpromazine (5.40, 11) | H | KR | Risperidone (2.10, 43) | M | CR | D | Moderate | No | 3 | -1.65 | -0.35 | 1.30 (7.00, 10.01) | 0.00 |

^a^ Ω_025,_ the signal value of the Ω Shrinkage measure model.

^b^χ, the signal value of the Chi-square statistics model.

^c^ CRR, Combination risk ratio.

^d^ PRR, proportional reporting ratio of drug D1 ∩ drug D2.

^e^χ^2^, Chi-squared of drug D1 ∩ drug D2.

^f^ M, QT-prolonging agents (Moderate Risk - Avoid) interacting drug in Lexicomp^®^ Risk Category.

^g^ KR, known risk of TdP in Lexicomp^®^ Risk Category.

^h^ I, QT-prolonging Agents (Indeterminate Risk - Avoid) interacting drug in Lexicomp^®^ Risk Category.

^i^ CR, conditional of TdP in Lexicomp^®^ Risk Category.

^j^ D, Consider therapy modification in Lexicomp^®^ Risk Rating.

^k^ NO, absence of Drug 1 and Drug 2 combinations in the open database of drug-related TdP case literature.

^l^ H, QT-prolonging agents (Highest Risk) interacting drug in Lexicomp^®^ Risk Category.

^m^ X, Avoid combination in Lexicomp^®^ Risk Rating.

^n^ Yes , presence of Drug 1 and Drug 2 combinations in the open database of drug-related TdP case literature.

^o^ C, Monitor therapy in Lexicomp^®^ Risk Rating.

^p^ L, QT-prolonging agents (Low Risk - Avoid) interacting drug in Lexicomp^®^ Risk Category.

^q^ B, No action needed in Lexicomp^®^ Risk Rating.

^r^ NA, uncategorized in CredibleMed^®^.

We evaluated the TdP by non-professionals for concomitant use of two of the drugs, totaling 1862 drug combinations, with 89 combinations being reported in at least 3 cases. Among these, 49 combinations were detected by all four statistical models, with 5 combinations indexed by Lexicomp^®^, 10 combinations indexed by Drugs.com^®^, 10 combinations indexed by either, 39 combinations indexed by neither, and only 5 combinations were indexed by both when considering all data, including reports from non-professionals. Sensitivity analyses revealed that the inclusion of non-professional reports did not alter the final outcome of the signal detection (Table 3-4).

**Table 3. DDI signal detection results containing data reported by non-professionals**

| Drug 1 (ROR, N) | Drug 1 Risk Category in Lexicomp^®^ | Drug 1 Risk Category in CredibleMeds^®^ | Drug 2 (ROR, N) | Drug 2 Risk Category in Lexicomp^®^ | Drug 2 Risk Category in CredibleMeds^®^ | Combination Risk Category in Lexicomp^®^ | Combination Risk Category in Drugs.com^®^ | Literature Database | Cases  (n_111_) | Ω_025_^a^ | χ^b^ | CRR^c^ (PRR^d^, χ^2e^) | Additive model^f^ |
| --- | --- | --- | --- | --- | --- | --- | --- | --- | --- | --- | --- | --- | --- |
| Amiodarone (48.53, 524) | H^g^ | KR^h^ | Fluoxetine (10.42, 234) | L^i^ | CR^j^ | C^k^ | Major | Yes^l^ | 33 | 1.89 | 10.88 | 5.53 (264.87, 8351.69) | 0.08 |
| Amiodarone (48.53, 524) | H | KR | Ciprofloxacin (11.45, 200) | I^m^ | KR | C | Major | Yes | 49 | 2.32 | 15.66 | 6.78 (324.58, 15314.61) | 0.10 |
| Amiodarone (48.53, 524) | H | KR | Fluphenazine (45.24, 29) | L | NA^n^ | C | Major | No^o^ | 22 | 4.03 | 32.75 | 55.81（2665.98, 55708.44) | 0.98 |
| Citalopram (13.24, 299) | M^p^ | KR | Quetiapine (5.88, 231) | H | CR | X^q^ | Major | Yes | 86 | 1.58 | 13.01 | 3.54（46.89, 3741.53) | 0.01 |
| Diltiazem(8.33, 119) | I | CR^l^ | Quetiapine (5.88, 231) | H | CR | C | Moderate | No | 21 | 2.21 | 11.25 | 10.92（90.71, 1766.58) | 0.03 |

**Table 4. DDI signal detection results excluding data reported by non-professionals**

| Drug 1 (ROR, N) | Drug 1 Risk Category in Lexicomp^®^ | Drug 1 Risk Category in CredibleMeds^®^ | Drug 2 (ROR, N) | Drug 2 Risk Category in Lexicomp^®^ | Drug 2 Risk Category in CredibleMeds^®^ | Combination Risk Category in Lexicomp^®^ | Combination Risk Category in Drugs.com^®^ | Literature Database | Cases  (n_111_) | Ω_025_^a^ | χ^b^ | CRR^c^ (PRR^d^, χ^2e^) | Additive model^f^ |
| --- | --- | --- | --- | --- | --- | --- | --- | --- | --- | --- | --- | --- | --- |
| Amiodarone (48.53, 524) | H^g^ | KR^h^ | Fluoxetine (10.42, 234) | L^i^ | CR^j^ | C^k^ | Major | Yes^l^ | 29 | 1.69 | 9.33 | 4.96 (235.37, 6494.10) | 0.07 |
| Amiodarone (48.53, 524) | H | KR | Ciprofloxacin (11.45, 200) | I^m^ | KR | C | Major | Yes | 46 | 2.23 | 14.60 | 6.45 (306.77, 13574.22) | 0.10 |
| Amiodarone (48.53, 524) | H | KR | Fluphenazine (45.24, 29) | L | NA^n^ | C | Major | No^o^ | 19 | 3.87 | 30.32 | 56.13（2664.12, 47759.00) | 0.98 |
| Citalopram (13.24, 299) | M^p^ | KR | Quetiapine (5.88, 231) | H | CR | X^q^ | Major | Yes | 81 | 1.48 | 13.98 | 3.40（44.15, 3311.00) | 0.01 |
| Diltiazem(8.33, 119) | I | CR^l^ | Quetiapine (5.88, 231) | H | CR | C | Moderate | No | 18 | 1.95 | 9.40 | 9.65（78.08, 1288.74) | 0.03 |

^a^Ω_025,_ the signal value of the Ω Shrinkage measure model.

^b^χ, the signal value of the Chi-square statistics model.

^c^CRR, Combination risk ratio.

^d^PRR, proportional reporting ratio of drug D1 ∩ drug D2.

^e^χ^2^, Chi-squared of drug D1 ∩ drug D2.

^f^Additive model, the signal value of the Additive model.

^g^ H, QT-prolonging agents (Highest Risk) interacting drug in Lexicomp® risk category.

^h^ KR, known risk of TdP in CredibleMed^®^ risk categor.

^i^ L, QT-prolonging agents (Low Risk - Avoid) interacting drug in Lexicomp® risk category.

^j^ CR, conditional of TdP in CredibleMed^®^ risk category.

^k^ C, Monitor therapy in Lexicomp® risk rating.

^l^ Yes, presence of Drug 1 and Drug 2 combinations in the open database of drug-related TdP case literature Literature database.

^m^ I, QT-prolonging Agents (Indeterminate Risk - Avoid) interacting drug in Lexicomp® risk category.

^n^ NA, uncategorized in CredibleMed^®^.

^o^ NO, absence of Drug 1 and Drug 2 combinations in the open database of drug-related TdP case literature Literature database.

^p^ M, QT-prolonging agents (Moderate Risk - Avoid) interacting drug in Lexicomp® risk category.

^q^ X, Avoid combination in Lexicomp^®^ Risk Rating.
